# Osteology of *Batrachuperus yenyuanensis* (Urodela, Hynobiidae), a high-altitude mountain stream salamander from western China

**DOI:** 10.1371/journal.pone.0211069

**Published:** 2019-01-25

**Authors:** Jia Jia, Jian-Ping Jiang, Mei-Hua Zhang, Ke-Qin Gao

**Affiliations:** 1 School of Earth and Space Sciences, Peking University, Beijing, China; 2 Key Laboratory of Economic Stratigraphy and Palaeogeography, Chinese Academy of Sciences (Nanjing Institute of Geology and Paleontology), Nanjing, China; 3 Chengdu Institute of Biology, Chinese Academy of Sciences, Chengdu, China; University of Akron, UNITED STATES

## Abstract

*Batrachuperus yenyuanensis*, commonly known as Yenyuan Stream Salamander, is a hynobiid species inhabiting high-altitude (2440–4025 m above sea level) mountain stream and pond environments along the eastern fringe of the Qinghai-Tibetan Plateau in western Sichuan Province, China. Although the species has been known for almost 70 years since its initial discovery in 1950, a thorough osteological description has never been provided. Our study provides a detailed account of the bony anatomy of this species, based on micro computed tomography scanning of multiple specimens collected from the type locality Shuangertang at Bailinshan, Yanyuan County, and several other localities in Sichuan Province. Our revised species diagnosis utilizes both bony and soft anatomical features. Comparative study of the specimens from the type locality in Yanyuan area with those from the nearby Xichang and Mianning areas confirms that they all pertain to *Batrachuperus yenyuanensis*, thereby removing doubt on the occurrence of the species in the latter areas. With this confirmation, the distribution of the species is extended from the type locality northwards some 180 km to the Mianning area, on both the west and east sides of the Yalong River, which is a major tributary of the upper Yangtze River. This distribution pattern indicates that the biogeographic origin and historical evolution of the species are closely associated with the orogeny of the Hengduan Mountains and formation of the Yalong River. Given the basalmost position of *Batrachuperus yenyuanensis* in relation to other congeneric species based on molecular studies, however, early expansion of the species distribution by dispersal is expected following the origin of the genus in early–middle Miocene in western Sichuan Province. Thus, the species may well have achieved its current distribution in western Sichuan before the drastic uplift of the Qinghai-Tibetan Plateau in Pliocene.

## Introduction

The family Hynobiidae (Amphibia: Urodela) has been widely accepted as a primitive group of salamanders [1–5]. Within the family, the genus *Batrachuperus* includes six or seven species that are all endemic to western China [6–8]. The type species, *Batrachuperus pinchonii* [9], has long been known from the Baoxing area, near Yaan in western Sichuan Province (see reference [6] for taxonomic history of the species). Approximately 400 km southwest of the Baoxing area, another species, *B*. *yenyuanensis*, was recorded in the literature over half a century ago (see below). The purposes of our paper are to provide a detailed osteological description of this species based on specimens processed by micro-CT scanning and clearing and double staining, and a discussion of the distribution patterns in relation to the biogeographic origin and evolution of the species in Sichuan Province.

**Fig 1:**
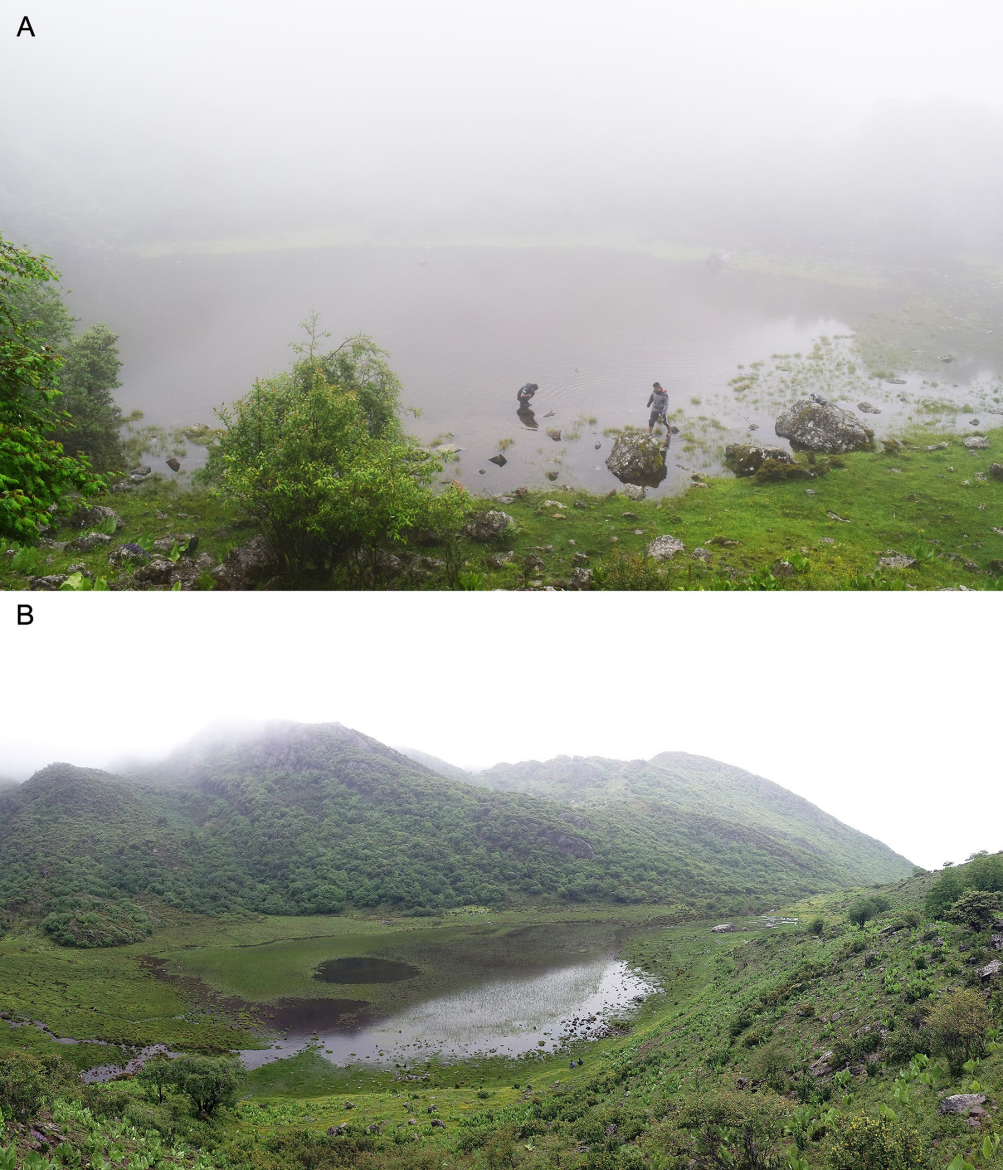
Photographs of the upper and lower ponds showing the high-altitude mountain pond and stream environments inhabited by *Batrachuperus yenyuanensis* at the type locality Shuangertang, near Yanyuan, Liangshan Yi Autonomous Prefecture, Sichuan Province, Southwest China. A, upper pond; B, lower pond (both looking to the west; photograph by J Jia).

*Batrachuperus yenyuanensis*, commonly known as the Yenyuan Stream Salamander, is of special interest because it inhabits high-altitude stream and pond environments along the eastern fringe of the Qinghai-Tibetan Plateau, at elevations ranging between 2440−4025 m above sea level (see below). The Yenyuan Stream Salamander was first named and described by the late Professor C-C. Liu [10] based on 60 specimens collected from the type locality Peilinshan (old spelling of the locality name; now spelled as Bailinshan, but we use “Shuangertang” as explained below), approximately 10 km southeast of the county town of Yanyuan, Liangshan Yi Autonomous Prefecture, Sichuan Province, China (Fig 1). The place named Shuangertang, meaning “twin ponds”, as used in our paper can be found in an official map of the area, whereas Peilinshan or Bailinshan refers to the mountain range bordering the Yanyuan County on the north and the Yanbian County on the south side of the mountain. Thus, using the terms Peilinshan or Bailinshan for the type locality can misleadingly refer to anywhere along either side of the mountain range. At the type locality Shuangertang, the two ponds are ~500 m apart, with one positioning at a higher elevation than the other. The specimens described in the original publication [10] were collected from both the lower and upper ponds, the altitudes of which were reported as 14500 ft. (~4420 m) and 15000 ft. (~4570 m) above sea level, respectively. However, these elevation data as obtained in 1940s are obviously incorrect, because the highest peak (Chuandongzi) of the Bailinshan Mountain is only ~4196 m above sea level (www.scyanbian.gov.cn). As presented elsewhere in our paper, elevation data collected by our own fieldtrip to Shuangertang in July of 2018 indicate that the lower pond at the type locality is actually positioned at 3902 m and the upper pond at 4025 m above sea level (data collected by J Jia).

Besides the type locality, the species is also known from several other sites in western Sichuan Province (S1 Table). With the exception of Xieka, a single locality in Jiulong County, Ganzi Tibetan Autonomous Prefecture, all other localities are within the territory of the Liangshan Yi Autonomous Prefecture (see discussion below). Occurrences of this species in the Xichang and Mianning areas were previously regarded as doubtful [6, 8, 11], but are confirmed by our study (see discussion below).

Phylogenetic studies based on molecular data have placed *Batrachuperus yenyuanensis* at a basal or the basal most position within the genus *Batrachuperus* [7, 12–16]. However, morphological features, especially the bony anatomy of the species, have remained poorly known since the initial publication of the species in 1950. Our paper represents one of a series of contributions documenting osteological details for each species in *Batrachuperus*. New information revealed in our study will help to clarify the relationships of the species within the genus and, more globally, the phylogeny of the Hynobiidae. However, we avoid conducting a phylogenetic analysis because the morphological details revealed in this study will be included in a work in progress on a combined analysis of the Hynobiidae. Instead, we provide a discussion on patterns of distribution and biogeographic evolution of the species in southwest Sichuan Province. It is our hope that this paper will stimulate further research to explain how the geologic history and formation of the current geo-topography in the area affected the distribution patterns of the species along the eastern fringe of the Qinghai-Tibetan Plateau.

## Materials and methods

A total of 34 specimens from six different localities were micro-CT scanned for our study (S1 and S2 Tables). The fluid-preserved (10% formaldehyde) specimens are deposited in the Chengdu Institute of Biology (CIB), Chinese Academy of Sciences, Chengdu, China. An Institutional Animal Care and Use Committee (IACUS) permit was obtained from the Animal Ethic Committee of the Chengdu Institute of Biology, Chinese Academy of Sciences (Permit Number: 20160305-AR).

Parameters of CT-scanning for specimens are provided as a supplementary table (S2 Table). Among the sampled specimens, FMNH 49371 is one of the paratype specimens for the species by original designation [10], whereas the holotype (FMNH 49370) could not be on loaned to us for micro-CT scan because of the risk of damaging the specimen. FMNH 49371 has a total length of ~175 mm, with a snout−vent length of ~80 mm. The specimen is interpreted as a subadult, because its articulars are not yet fully ossified as revealed by micro-CT scan (see [17, 18] for discussion). Other specimens used in the study include both young and fully-grown adults based on the extent of ossification of their articulars and mesopodial elements (see reference [18] for discussion on ossification of the articular in hynobiids).

FMNH 49371 was micro-CT scanned using a dual tube X-ray scanner (GE phoenix v/tome/x 240kv/180kv, Boston, USA) at the PaleoCT Laboratory, University of Chicago. All other specimens were scanned using a scanner (Quantum GX micro-CT Imaging System, PerkinElmer, Waltham, USA) at the Chengdu Institute of Biology, Chinese Academy of Sciences. In addition, three specimens (CIB 201707YY04, 201707YY08, 201707YY09) from the lower stream of the type locality Shuangertang (Peilinshan or Bailinshan) at an elevation of ~3000 m above sea level were cleared and double stained, following the protocol described in [19]. Among these, CIB 201707YY04 is a post-metamorphic juvenile (TL = 113.34 mm) with cartilaginous articulars and incomplete ossification of limbs; CIB 201707YY08 is a male adult (TL = 169.48 mm) with well-ossified articulars and mesopodia in the limbs; and CIB 201707YY09 is a female young adult (TL = 154.78 mm) with partially ossified articulars and incomplete ossification of the mesopodia.

The cranium of three specimens (FMNH 49371, CIB 17308, 72599) were segmented and rendered by using the software package VG Studio Max 2.2 (Volume Graphics, Heidelberg, Germany). The specimens were measured in millimeters (mm) by hand-hold calipers and via the measurement module implanted in VG Studio Max 2.2. Total length (TL) refers to the measurement between the tip of the snout to the posterior extremity of the tail; snout−vent length (SVL) is the measurement from the tip of the snout to the opening of the cloaca. Because the cloaca opens below the mid-length of the last caudosacral, the term snout−pelvic length (SPL) used in reference to the skeleton refers to the measurement from the tip of the snout to the end of the last caudosacral vertebra. Skull length (SKL) refers to the maximum dimension from the tip of the snout to the posterior end of the occipital condyles; skull width (SKW) is the distance measured across the cranio-mandibular joint.

**Institutional abbreviations**—**CIB**, Chengdu Institute of Biology, Chinese Academy of Sciences, Chengdu, Sichuan Province, China; **FMNH**, Field Museum of Natural History, Chicago, Illinois, USA.

**Anatomical abbreviations**—**act**, acetabulum; **adf**, anterodorsal fenestra; **amf**, anteromedial fenestra; **an**, angular; **anf**, angular foramen; **ar**, articular; **at**, atlas; **bb,** basibranchial; **bc**, basale commune; **c**, centrale; **cb,** ceratobranchial; **ch**, ceratohyal; **cho**, choana; **cora**, coracoid; **corn**, cornua; **crd**, crista dorsalis; **crv**, crista ventralis; **dc**, distal carpal; **den**, dentary; **dt**, distal tarsal; **facf**, foramen faciale; **fcuv**, fossa cubitalis ventralis; **fe**, femur; **fi**, fibula; **fib**, fibulare; **foid**, inferior dental foramen; **fom**, mental foramen; **fopt**, foramen opticum; **fpal**, foramen palatinum; **fpot**, foramen post-oticum; **fr**, frontal; **glf**, glenoid fossa; **hb**, hypobranchial; **hu**, humerus; **i**, intermedium; **icf**, internal carotid foramen; **il**, ilium; **isc**, ischium; **lac**, lacrimal; **mmk**, mentomeckelian; **mx**, maxilla; **na**, nasal; **obf**, obturator foramen; **obs**, orbitosphenoid; **op-ex**, opisthotic-exoccipital complex; **pa**, parietal; **pm**, premaxilla; **po**, postminimus; **pra**, prearticular; **prco**, procoracoid; **prf**, prefrontal; **pro**, prootic; **ps**, parasphenoid; **pt**, pterygoid; **pub**, pubis; **qu**, quadrate; **ra**, radius; **rad**, radiale; **rl**, radial loop; **sca**, scapulocoracoid; **scof**, supracoracoid foramen; **sm**, septomaxilla; **spsc**, suprascapular; **sq**, squamosal; **st**, stapes; **ti**, tibia; **tib**, tibiale; **tro**, trochanter; **troc**, trochanteric crest; **ul**, ulna; **uln**, ulnare; **vo**, vomer; **y**, element y; **yps**, ypsiloid cartilage.

## Results

### Systematics and description

Order Urodela Duméril, 1806 [20]

Suborder Cryptobranchoidea Dunn, 1922 [1]

Family Hynobiidae Cope, 1859 [21]

Genus *Batrachuperus* Boulenger, 1878 [22]

Species *Batrachuperus yenyuanensis* Liu, 1950 [10]

**Holotype**: FMNH 49370, a male adult with a TL of 168 mm and a SVL of 79 mm (original type designation in reference [10]). The specimen was reported in the original publication as having been collected from the lower pond at 14500 ft. (~4420 m) above sea level, but is corrected here as 3902 m above sea level (see Introduction above).

**Paratype**: FMNH 49371, a male adult with a TL of ~175 mm (one of the 59 paratype specimens by original designation in C-C. Liu [10]). The specimen was reported as having been collected from the upper pond at 15000 ft. (~4570 m) above sea level, but is corrected here as from 4025 m above sea level (see Introduction above).

**Type locality**: Peilinshan, Yenyuan County, Sikang, as in the original publication by C-C. Liu [10]; at present, the mountain range is spelled as Bailinshan and the actual locality is called Shuangertang (N27°20' 319", E101°32' 253"); the locality is approximately 10 km southeast of the county town of Yanyuan, Liangshan Yi Autonomous Prefecture, Sichuan Province, China.

**Revised diagnosis**: Skull slightly longer than wide (versus as wide as long in *B*. *karlschmidti*); anterodorsal fenestra one-third to one-half the size of the narial opening; otic process of squamosal well developed (vs. absent in *B*. *tibetanus* + *B*. *karlschmidti* + *B*. *cochranae*); vomerine teeth four to six in number in each tooth row, with rows arranged as two short arcs, bowed anteriorly and widely separated from one another (vs. straight rows in *B*. *londongensis*); a single mental foramen opens within anterior one-fourth of the dentary (shared with *B*. *taibaiensis*); basibranchial II ossified, inverted T-shaped; presacral vertebrae 17 in number (vs. 18 in *B*. *londongensis* and *B*. *taibaiensis*, 16 in *B*. *cochranae*); retention of two centralia in fore- and hind limbs; distinct longitudinal skin folds present ventrally between mandibles; tail longer than snout−vent length, with a tail fin developed as a thin crest (vs. tail in same length or shorter than snout−vent length and thick tail fin in other congeners); palmar tubercles present in both the manus and pes; terminal phalanx covered with a cornified epidermal sheath.

**Known distribution and habitat**: *Batrachuperus yenyuanensis* is confined to southwestern Sichuan Province, China. Besides the type locality Shuangertang at Bailinshan, the species also occurs in mountain streams in Xichang and Mianning areas (see later discussion). The salamander lives in high-altitude mountain streams and ponds at elevations up to 4025 m above sea level in the type Yanyuan area (Fig 1), but reaches a lower elevation of 2440 m above sea level in the Mianning area. The salamander is nocturnal, as adults can often be found hiding under rocks in water during daytime. Adults feed mainly on freshwater shrimp and insects, but their diet also includes plant seeds and algae [6, 8, 10].

**Fig 2:**
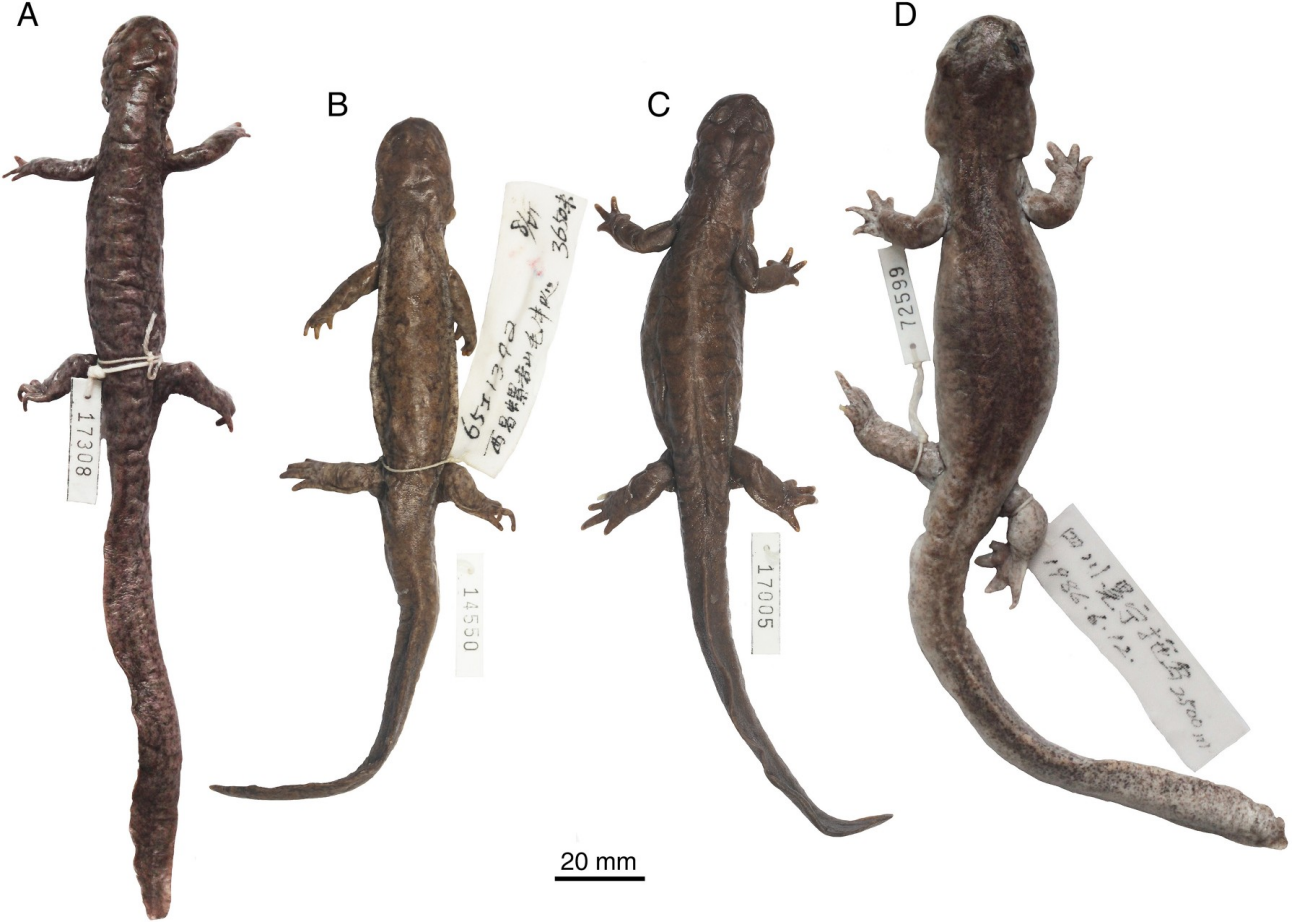
Photographs of representative formaldehyde-preserved specimens of *Batrachuperus yenyuanensis* in dorsal view. A, CIB 14308 from the type locality Shuangertang, Yanyuan County; B, CIB 14550 from Maoniudui, Dechang County; C, CIB 17005 from Shenguozhuang, Yuexi County; D, CIB 72599 from Tuowu, Mianning County.

**External morphology:** Adults have a TL ranging between 144−231 mm. The skull is flat, longer than wide, with a short and rounded snout (Fig 2). Distinct longitudinal skin folds are present on the ventral aspect of the head between the mandibles. Labial folds are well developed, partly covering the lower lips; gular folds are present on either side of the neck. Costal grooves, 11 or 12 in number, are developed along each side of the trunk region. There are four digits in both fore- and hind limbs, with the terminal phalanges covered with dark cornified sheath. The tail is longer than the SVL, with a dorsal fin fold of the tail developed as a thin crest [6, 10, 11]. Movable eye lids are present and sclerotic cartilages are lacking.

## Osteological description

### Dermal skull roof

**Fig 3:**
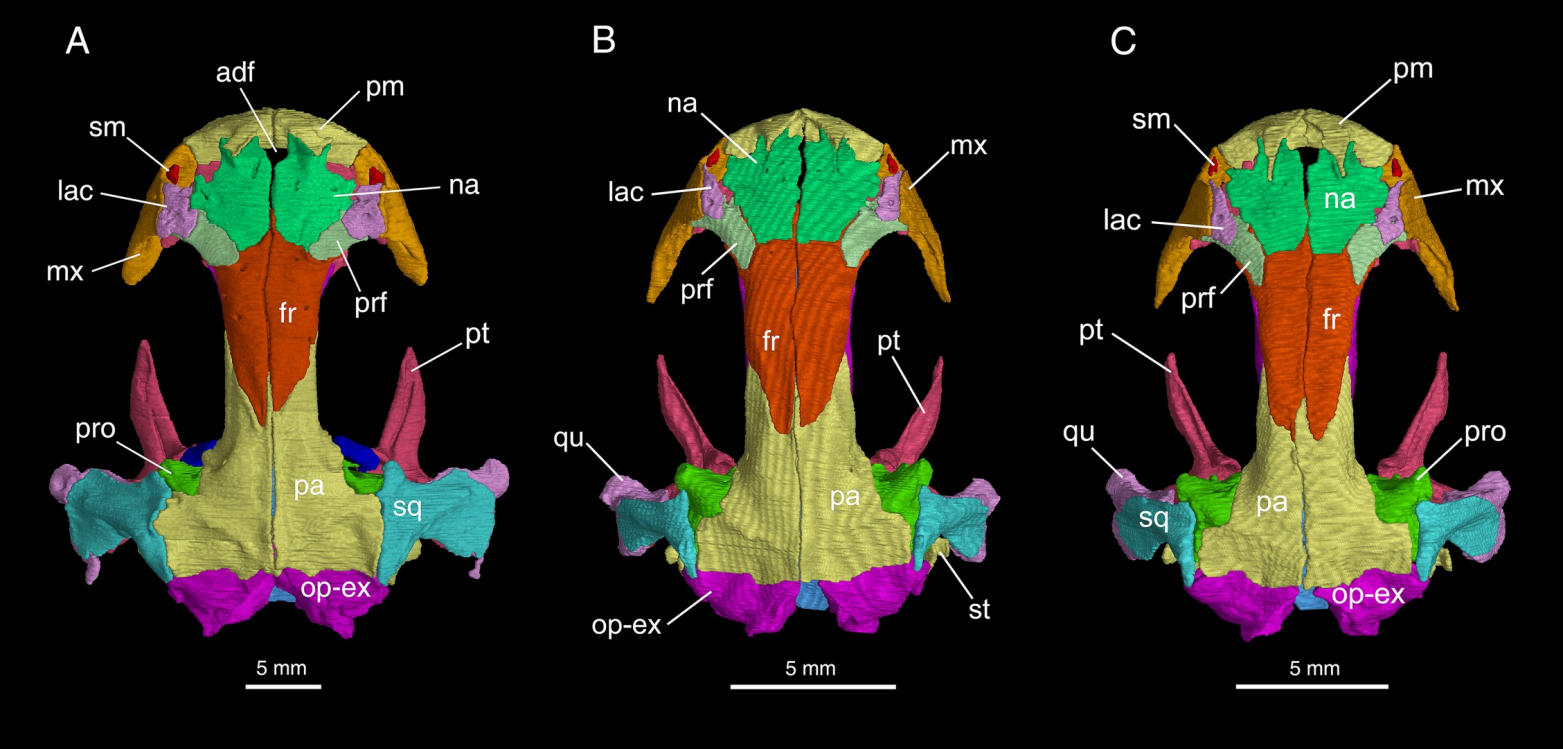
Micro-CT rendered reconstruction of the skull of *Batrachuperus yenyuanensis* in dorsal view. A, CIB 72599 from Tuowu, Mianning County; B, FMNH 49371, paratype specimen from the type locality Shuangertang, Yanyuan County; C, CIB 17308 from Shuangertang, Yanyuan County.

**Fig 4:**
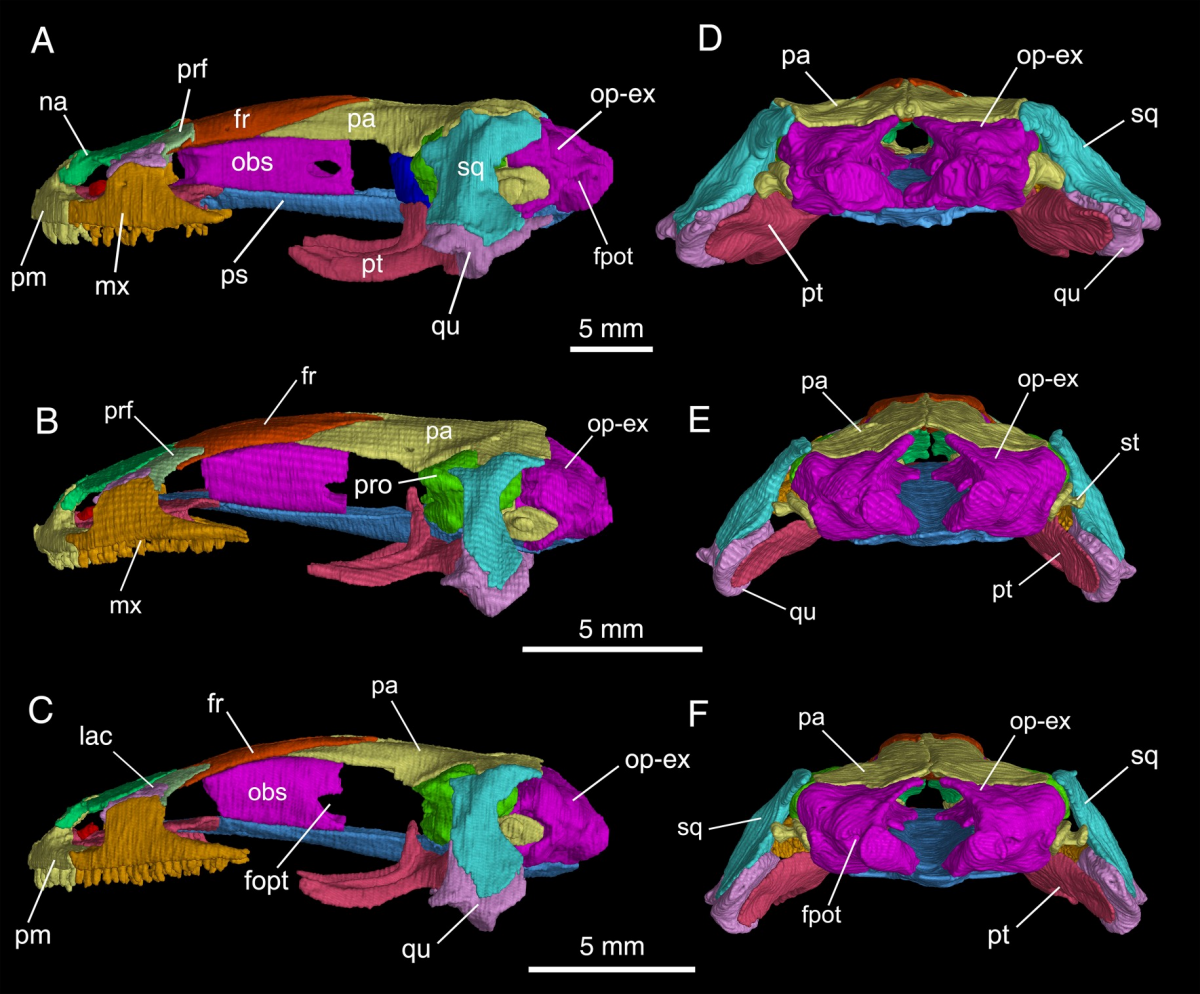
Micro-CT rendered reconstruction of the skull of *Batrachuperus yenyuanensis* in left lateral and occipital views. A, D, CIB 72599 from Tuowu, Mianning County; B, E, FMNH 49371, paratype specimen from Shuangertang, Yanyuan County; C, F, CIB 17308 from Shuangertang, Yanyuan County.

**Fig 5:**
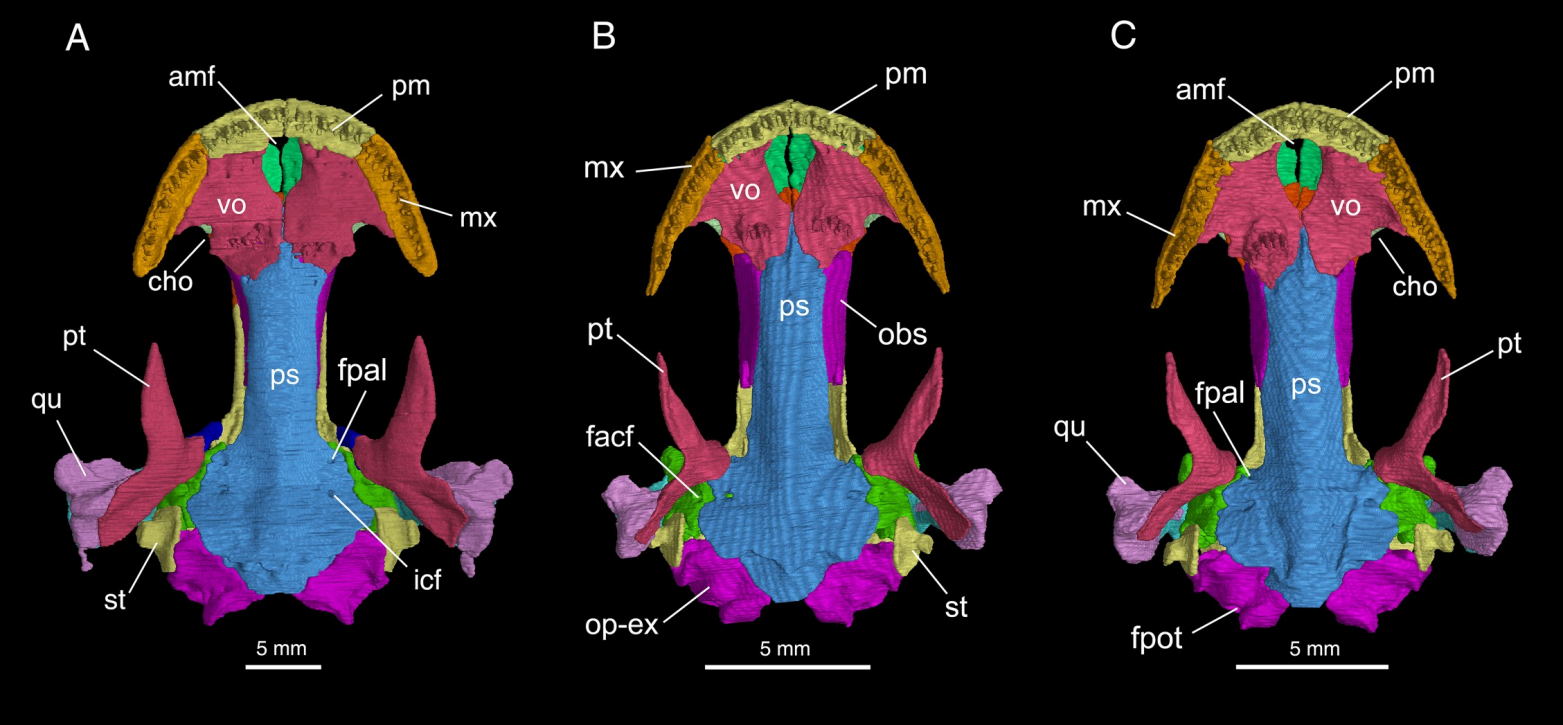
Micro-CT rendered reconstruction of the skull of *Batrachuperus yenyuanensis* in palatal view. A, CIB 72599 from Tuowu, Mianning County; B, FMNH 49371, paratype specimen from Shuangertang, Yanyuan County; C, CIB 17308 from Shuangertang, Yanyuan County.

The skull is slightly longer than wide, with a short and rounded snout (Fig 3). The premaxillae are paired, each bearing a spike-like pars dorsalis (alary process) overlapping the nasal. Between the partes dorsalis opens the anterodorsal fenestra (premaxillary fontanelle of [23]), which is one-half to one-third the size of the external naris (see discussion below). Laterally at the base of the pars dorsalis, a small foramen opens into the naris for passage of a terminal twig of the mesial branch of the ramus ophthalmicus profundus (CN V^1^). Along the ventral border of the narial opening, the premaxilla extends laterally to meet with the maxilla. On the lingual side, the pars palatina of the premaxilla is a narrow shelf contributing to a small part of the palate (see below). Ventral to the shelf, the pars dentalis of the premaxilla bears a short tooth row, containing no more than eleven teeth (Figs 4A–4C and 5).

The paired nasals are large bony plates roofing the nasal capsules immediately posterior to the narial openings. The two nasals meet along a straight midline suture, but are anteromedially notched for the border of the anterodorsal fenestra, which forms the dorsal opening for the intermaxillary or internasal gland [24]. The nasals are bilaterally expanded to the extent that they are slightly wider than the frontals, a plesiomorphic feature seen in all hynobiids [1]. The dorsal surface of the nasal is penetrated by several small foramina (foramina mediale nasi in reference [24]), through which pass the ultimate twigs of the mesial branch of the ramus ophthalmicus profundus (CN V^1^). The nasal laterally contacts the lacrimal and prefrontal, but not with the dorsal process of the maxilla. Posteriorly, the nasal slightly overlaps the frontal, but dorsally displays a transverse or oblique suture with the frontal at a level just posterior to the anterior border of the orbits (Fig 3; S1A–S1C Fig).

The paired frontals are elongate plates that meet one another along a straight midline suture. The anterior part of the frontal widens and thins out to underlay the nasal and prefrontal. In the interorbital region, the frontals have parallel lateral margins that furnish the anterior one-half of the medial border of the orbit, whereas the posterior one-half of the orbit is provided by the parietal. The lateral margin of the frontal curves ventrally to articulate with the orbitosphenoid and a small part of the parietal (see below). The posterior one-third of the frontal narrows towards the midline to form a tongue-like process overlapping the parietal.

The paired parietals form a large table roofing the orbito-temporal region of the skull. The anterior extension of the table displays parallel lateral margins, contributing to the posterior half of the orbital border (Figs 3 and 4A–4C). Overlapped by the tongue-like process of the frontal, this part of the parietal is dorsolaterally exposed as a short anterolateral process wedged between the frontal and orbitosphenoid. This process extends only to the midlevel of the orbit, thus failing to contact the prefrontal anteriorly. Dorsally, at the posterior border of the orbit, the parietal swings laterally to form a small triangular process that slightly curves downward to contact the prootic. This process is perforated by a small foramen for passage of the nervus trochlearis (CN IV), as has been identified in other salamanders [24–26]. Posterior to this process, the parietal table widens laterally to form a boot-like extension in articulation with the squamosal in fully grown adults (e.g., CIB 17308, 17310, 17313, 72599; Fig 3A–3C). However, several subadults (e.g., FMNH 49371, CIB 17302, 17309, 17314) display a narrow gap between the parietal and squamosal (Fig 3B and 3C; S1A–S1C Fig). The dorsal surface of the parietal table slopes posteriorly to form a depression (cervical epaxial fossa), where the M. spinalis capitis is attached [27, 28]. The posterior border of the parietal displays a transverse suture with the opisthotic-exoccipital complex (Figs 3 and 4D–4F).

The maxilla is short and lightly built, with its pars dorsalis (facial process) rising to form the lateral wall of the nasal cavity (Fig 4A–4C; S2 Fig). The facial process is anteriorly notched for the narial opening and posteriorly notched for the orbit. The dorsal rim of the process contacts with both the lacrimal and prefrontal, but has no contact with the nasal. The lateral surface of the facial process is penetrated by small foramina (foramina laterale nasi), through which pass the lateral branch of the ramus ophthalmicus profundus (CN V^1^) to innervate the skin of the face [24]. As observed in palatal view, the pars palatina of the maxilla is a narrow shelf contributing to the lateral part of the palate. The shelf becomes extremely narrow posteriorly and vanishes at the posterior end of the tooth row. Roughly at the mid-level of the pars palatina, the inner side of the maxillary wall above the shelf displays a deep groove, which anteriorly leads to the infraorbital canal for passage of the superior alveolar branch of the nervus trigeminus (CN V^1^) and associated vessels [24]. The posterior process of the maxilla is short, terminating at a point slightly anterior to the midlevel of the orbit. The pars dentalis of the maxilla bears 18−20 teeth, with the tooth row ending at a point close to the posterior extremity of the bone.

The septomaxilla in all adult specimens is ossified as a small bone within the narial opening (Fig 3; S2 Fig). The septomaxilla is irregular in shape, with its inner side strongly scrolled to form a tube (pars tubularis) for passage of the nasolacrimal duct [29]. A small medial process is observable in most specimens, projecting posterodorsally from the pars tubularis. Our observation of comparative specimens indicates that all species of *Batrachuperus* have the septomaxilla ossified at the adult stage. A septomaxilla is commonly seen in all hynobiids, in contrast to its absence in extant cryptobranchids [17, 28, 30].

The lacrimal is a bony plate set between the nasal and maxilla. In many specimens of small to medium size (i.e., SVL less than 200 mm), the lacrimal is narrow anteriorly, wider posteriorly, and more or less triangular in shape (e.g., FMNH 49371, CIB 17310, 17313); the lacrimal in these specimens extends anteriorly to enter the border of the narial opening, but posteriorly either enters or fails to enter the border of the orbit (Fig 3; S1A–S1C Fig). In large specimens with a total length close to or over 200 mm (e.g., CIB 17308, 72594, 72595, 72597, 72599), more extensive ossification of the lacrimal over the prefrontal results in an “L”-shaped configuration of the bone, and it enters the naris only or enters both the naris and orbit (Fig 3A; S1A–S1C Fig; see later discussion). Whether the lacrimal enters the border of the orbit or not, a large lacrimal foramen opens on the dorsal surface of the bone, with a short nasolacrimal canal running obliquely through the bony plate and opening anteroventrally within the naris. A distinct lacrimal is present in most hynobiids but *Hynobius formosanus*, in which the lacrimal is fused completely with the prefrontal [31].

The prefrontal is an elongate plate, set in an oblique position at the anteromedial border of the orbit (Fig 3; S1A–S1C Fig). It articulates with the nasal and frontal medially, and meets the facial process of the maxilla laterally beneath the lacrimal. Much of its lateral border forms the anterior rim of the orbit together with the lacrimal and maxilla. Posteriorly, a prefrontal-parietal contact is absent as commonly seen in other hynobiids, but in contrast to the condition in cryptobranchids, in which the two bones meet one another [28, 32].

### Suspensorium

The squamosal is roughly a “T”-shaped structure, with a short crossbar parallel to, and an elongate stem perpendicular to the sagittal plane of the skull. The crossbar sutures with the parietal, whereas the stem slopes ventrolaterally towards the cranio-mandibular joint (Figs 3 and 4; S1A–S1C Fig). Anteriorly, the crossbar bears a prominent otic process overlapping the alar process of the prootic; a much larger posterior process overlaps a part of the opisthotic-exoccipital complex. The stem of the squamosal is dorsally convex, but ventrally concave to receive the ascending process of the quadrate. The distal end of the stem widens posteriorly to form a triangular process, which serves as the origin of the M. interhyoideus posterior, a hyobranchial levator that fans ventromedially below the hyoid arches [24, 33].

The quadrate is widened distally, where it is exposed beyond the squamosal, but a large part of the bone remains concealed beneath the squamosal (Figs 3 and 4; S1A–S1C Fig). Micro-CT scans of several specimens (e.g., FMNH 49371, CIB 17308, 17310, 17314, 72599) reveal that a large ascending process extends from the widened distal end towards the lateral boot of the parietal (S2A, S2C and S2E Fig). This ascending process is connected with the stylus of the stapes (columella) by the ligamentum squamoso-columellare, which may also connect with the squamosal [34]. At the widened distal end of the quadrate, a large, flap-like process projects anteriorly to overlap the quadrate process of the pterygoid, whereas a more robust process is directed anterolaterally. The expanded distal end is penetrated by a small quadrate foramen for the passage of the posterior condylar artery and vein [35]. The expanded distal end of the quadrate has a short posterior process (most prominent in the largest specimen CIB 72599), probably for connection with the ceratohyal by the ligamentum hyo-suspensoriale [34].

The pterygoid is tri-radiate and lightly built. Its palatal and quadrate processes are about the same length, but diverge at right angles. The palatal process is anterolaterally directed, terminating with a pointed tip for a ligamentous connection with the maxilla. The palatal process is ventrally smooth, but is dorsally grooved to receive a slender cartilaginous rod (processus pterygoideus of the palatoquadrate), which stems from the quadrate and extends along the entire length of the palatal process of the pterygoid. In the largest specimen so far known for the species (CIB 72599), the dorsal groove on the left pterygoid has its lateral walls strongly scrolled to form a partly closed tube (Fig 3A; S3A Fig). In palatal view, the otic process is short and obtuse, directed medially. In dorsal and lateral views, however, the anterior border of the otic process is strongly scrolled posterolaterally to hold the pillar-like ascending process of the palatoquadrate (Figs 3A and 4A and 4B). The otic process contacts neither the prootic nor the parasphenoid (Fig 5). The quadrate process of the pterygoid is a flat plate, dorsally articulating with the quadrate to reinforce the suspensorium (Figs 4E, 4F and 5; S1D–S1F Fig).

Dorsal to the otic process of the pterygoid, a pillar-like structure represents the perichondral ossification of the ascending process of the palatoquadrate [24] (but epipterygoid in reference [17]; metapterygoid in [36]) as seen in some other salamanders [24, 26]. This bony pillar can only be seen in those fully-grown adults (e.g., CIB 72599; Fig 4A; S2A and S2B Fig), whereas it is incompletely or not ossified at all in many adult or young adult specimens (e.g., CIB 17002, 17308, 72595; Fig 4B and 4C; S2C–S2F Fig). This suggests that ossification of the element is ontogenetically delayed until well after maturity.

### Palate and braincase

The partes palatina of the premaxilla and maxilla are narrow flanges that both have a sutural contact with the vomer. The postchoanal part of the maxillary flange, however, is diminished posteriorly and vanishes at the end of the maxillary tooth row. The vomers, the principal palatal elements, are large plates, irregular in shape, covering most of the palate between the choanae (internal nares). Anteriorly between the vomers, the anteromedial fenestra is a large and roughly oval-shaped opening for the intermaxillary or internasal gland, opposite to the anterodorsal fenestra described above (Fig 5; S1D–S1F Fig). The vomers meet medially for a short distance behind the fenestra, but diverge again posteriorly, with their posterior processes (posterior shelf of the vomer in reference [17]; parasphenoid process in [28]) overlapping the parasphenoid in palatal view. The posterolateral border of the vomer is notched for the choana, but a retrochoanal (postchoanal) process is absent: there is no laterally extending process immediately behind the choana. The anterior part of the vomer is penetrated by several small foramina, through which pass the ramus ventralis of the nervus trigeminus (CN V^1^) and the ramus palatinus of the nervus facialis (CN VII) to innervate the skin lining (membrane) of the palate [24].

The vomerine teeth, four to six in number in each of two tooth rows, are arranged to form a short arc that slightly bows anteriorly (Fig 5; S1D and S1F Fig). The tooth rows, widely separated from one another, are located medial to the choanae, posterior to the mid-length of the vomerine plate. A similar pattern is commonly seen in other congeneric species, except for *Batrachuperus londongensis*, in which the tooth row is basically straight and extends antero-posteriorly in its orientation [26].

Only the anterior part of the parasphenoid is involved in the formation of the palate, where it is exposed between the posterior processes of the vomers. The orbito-temporal region of the parasphenoid has parallel lateral borders, which curve dorsally in articulation with the orbitosphenoids (see below). More posteriorly, the basal plate of the parasphenoid is expanded, bringing its lateral side into contact with the prootic. Each of the lateral alae is perforated by an internal carotid foramen, through which passes the internal carotid artery [24]. Anterior to the internal carotid foramen, the parasphenoid is often penetrated by another small foramen (Fig 5A), the ventral opening for the passage of the ramus palatinus of the nervus facialis (CN VII) coming out from the foramen palatinum of the prootic. Posterior to the lateral alae, the ventral surface of the parasphenoid is often ridged to form side-by-side double depressions, which mark the attachment of the subvertebral muscles, including M. rectus cervicis [24, 28]. The parasphenoid terminates posteriorly in a large posteromedian process, which floors the foramen magnum between the opisthotic-exoccipital complexes.

The orbitosphenoid is a vertical plate, roughly rectangular in shape. The element forms the bony wall of the braincase in the orbito-temporal region. It articulates dorsally with the frontal and parietal, ventrally with the parasphenoid and in some specimens (e.g., FMNH 49371, CIB 17305, 17310, 72595) it may articulate with the vomer as well (Figs 4A–4C and 5; S1E and S1F Fig). As revealed by micro-CT scan of specimens, the orbitosphenoid bears a short anterovental process that curves medially to overlap the parasphenoid (S3 Fig). Anterior to the orbitosphenoid, the orbitonasal fenestra opens in the ethmoidal region; posterior to the orbitosphenoid is another large fenestra, through which passes the nervus oculomotorius (CN III). With exception of an abnormal condition on the left side in CIB 72599, all specimens show that the posterior border of the orbitosphenoid is deeply notched for the foramen opticum (cf. Fig 4A vs. 4B and 4C). The same patterns for these cranial nerves also occur in other extant hynobiids and their Early Cretaceous relatives [18].

The prootic forms the anterolateral wall of the otic capsule between the large fenestra for the nervus oculomotorius and the fenestra ovalis (Fig 4A and 4C; S2 Fig). Dorsally, the alar process (optic process) of the prootic is short, but quite robust. The process projects anterodorsally in lateral view, but anterolaterally in dorsal view. This process is dorsally exposed in a V-shaped cleft between the parietal and squamosal anterior to the sutural contact of the latter two elements (Fig 3). Ventrally, the inferior process (basal process) of the prootic is extremely short and knob-like, and ventrally sets in a small fossa of the otic process of the pterygoid. In ventral view, a small foramen palatinum opens ventral to the inferior process of the prootic for passage of the ramus palatinus of the nervus facialis (CN VII). This foramen can be observed in palatal view in several juvenile and subadult specimens (e.g., CIB 94627, 94632, CIB 201707YY02; S1D–S1F Fig), but is covered by the alae of the parasphenoid in large specimens, in which the foramen palatinum confluents with a canal that penetrates the alae of the parasphenoid (Fig 5). Posterolateral to the foramen palatinum opens a much larger foramen faciale for passage of the truncus hyomandibularis of the nervus facialis (CN VII). The posterior border of the prootic is notched for the anterior rim of the fenestra ovalis.

The stapes is ossified. It consists of a large disc-like footplate covering the fenestra ovalis and an extremely short stylus fused with the footplate (Figs 4 and 5; S2 Fig). The blunt distal end of the stylus is for a ligamentous connection (ligamentum squamoso-columellare in reference [37]) with the quadrate and/or squamosal. There is no trace of a stapedial foramen, in contrast to *Batrachuperus londongensis*, in which the foramen is present in some, but not all individuals [26]. No operculum, either bony or cartilaginous, is identified in any specimens of *B*. *yenyuanensis* that we examined. Micro-CT scans of specimens of subadults show a square gap immediately ventral to the fenestra ovalis between the prootic and opisthotic-exoccipital complex. In large and fully grown adults, however, this gap tends to be closed by more extensive ossification of the prootic and opisthotic-exoccipital complex (e.g., CIB 72599; Fig 5A).

Posterior to the fenestra ovalis is the fused opisthotic-exoccipital complex, which covers the posterolateral aspect of the otic capsule and borders the foramen magnum. Dorsally in articulation with the parietal and ventrally with the parasphenoid, the opisthotic-exoccipital complex is swollen posterolaterally to form the housing of the auditory capsule. Posteriorly, the exoccipital part of the complex bears paired occipital condyles for articulation with the cotyles of the atlas. Slightly lateral to the base of the occipital condyle, a small foramen (foramen post-oticum in reference [24]; foramen jugulare in reference [25]) opens for passage of the combined nervus glossopharyngeus and vagus (CN IX and X; Figs 4 and 5). The opisthotic-exoccipital complex does not meet its counterpart above the foramen magnum; instead, there is a small gap filled by the cartilaginous tectum synoticum.

### Mandible

**Fig 6:**
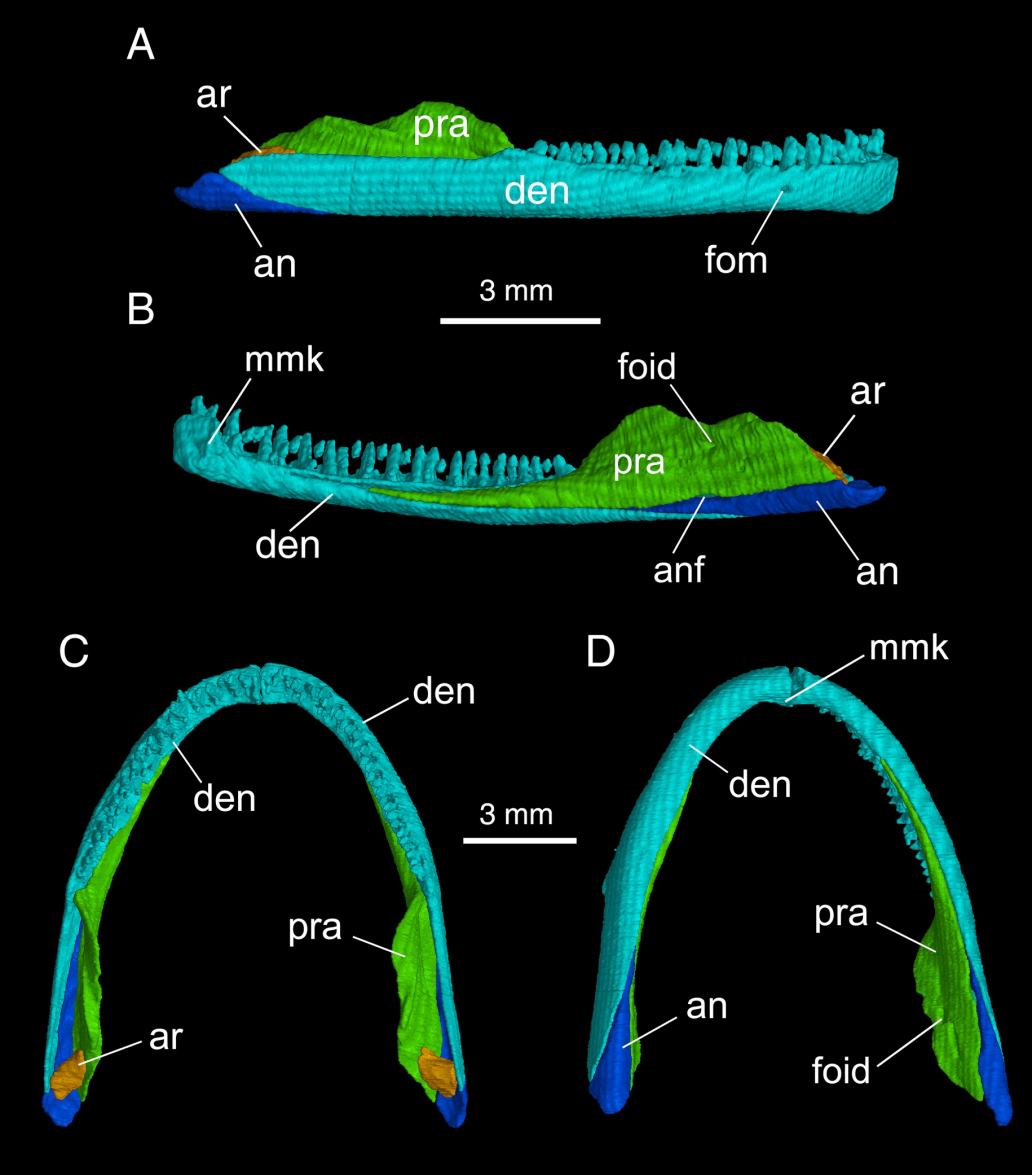
Micro-CT rendered reconstruction of the mandible of *Batrachuperus yenyuanensis*. A, right mandible in lateral view; B, right mandible in medial view; C, mandibular arch in dorsal view; D, mandibular arch in ventral view. FMNH 49371, paratype specimen, from Shuangertang, Yanyuan County.

The mandible consists of four bony elements: the dentary, prearticular, angular, and articular (Fig 6A–6D). In addition, complete fusion of a mentomeckelian with the dentary is indicated by the presence of a posterior mental process at the symphysis (Fig 6B–6D).

The dentary is a large bone, covering most of the lateral aspect of the mandible, and is the only tooth-bearing element in the lower jaw. The external surface of the dentary is slightly dorsoventrally convex, and bows laterally along the curvature of the mandibular ramus. The dentary is perforated by a single mental foramen within the anterior one-quarter of its length; a cutaneous branch of the ramus mandibularis (CN V_3_) passes through this foramen to innervate the skin muscle of the jaw. Presence of a single mental foramen in the mandible has been consistently observed in all the specimens we examined for this species, hence, is identified as a diagnostic feature distinguishing *B*. *yenyuanensis* from most other species of *Batrachuperus*, with the exception of *B*. *taibaiensis* (CIB 20040235) being the only congener sharing this feature with *B*. *yenyuanensis*.

The dentary carries 22–24 teeth, with the tooth row extending from the symphysis posteriorly to the point where the coronoid process of the prearticular begins its ascent (Fig 6A and 6B). In lateral view, the dentary terminates posteroventrally in a triangular process below the cranio-mandibular joint. This posterior process contacts the articular medially and the angular ventrally.

The prearticular, the second large bone of the mandible, covers most of the inner side of the jaw to close the Meckelian canal. The anterior process of the prearticular is an elongate tongue that extends anteriorly and terminates at a point slightly anterior to the mid-level of the dentary tooth row (Fig 6A). At the posterior end of the dentary tooth row, the prearticular rises to form a prominent coronoid process for insertion of the M. levator mandibulae. The process descends posteriorly from its apex and merges with a dorsal crest of the same bone; this forms the medial border of a deep trough, in which sits the articular of the cranio-mandibular joint. Posteroventral to the apex of the coronoid process, a deep horizontal groove leads to a foramen (inferior dental foramen in reference [24]) for passage of the ramus alveolaris of the nervus facialis (CN VII) and the alveolar artery. Anteroventral to the inferior dental foramen, the ventral border of the prearticular is slightly notched to accommodate an angular foramen (Fig 6B; see below).

At the cranio-mandibular joint, the articular is ossified in adults in a trough formed by the prearticular and dentary. In many adult specimens, the articular is ossified as a short stub (e.g., FMNH 49371; CIB 17305, 17308, 17310), whereas in the largest specimens (e.g., CIB 72599), it is more extensively ossified and elongated to approach the level of the anterior end of the palatal process of the pterygoid (S3A Fig). The articular is slender anteriorly, but is expanded posteriorly with a convex dorsal surface for articulation with the quadrate. Micro-CT scan of specimens reveals that the articular is ventrally penetrated by a small foramen, the articular foramen, for passage of the truncus hyomandibularis of the nervus facialis (CN VII). Based on our knowledge of extant hynobiids and their Early Cretaceous relatives, ossification of the articular can be used as a clear indication of sexual maturity [17, 18].

The angular is a slender splint, posteroventrally wedged between the prearticular and dentary. It is mostly exposed in ventral and medial views, but also has a small exposure in the lateral surface of the jaw. In ventral view, the angular narrows anteriorly and pinches out at the level of the coronoid process of the prearticular. Close to this point, a small angular foramen (Fig 6A; S1E and S1F Fig) opens between the prearticular and angular for a branch of the nervus trigeminus (CN V_3_). Presence of the angular as a separate element in the mandible is a plesiomorphic condition in all cryptobranchoids (i.e., hynobiids and cryptobranchids), whereas it fuses with the prearticular in salamandroids [1] (see references [38, 39] for discussion).

### Dentition

Tooth structure and crown patterns are observed using microscope, because micro-CT scans of the skull and whole-body specimens show no details of tooth structures. Both marginal and palatal teeth are pedicellate with the bicuspid crowns separated from the basal pedicels by a poorly mineralized dividing zone (S4 Fig) as commonly seen in other hynobiids. All marginal teeth are pleurodont, whereas vomerine teeth are ankylosed on ventral surfaces of the vomers. Resorption pits and replacement teeth are observable in specimens examined (S4A and S4C Fig). The premaxillary tooth row is short, containing 10 or 11 teeth as counted from several specimens, including the paratype (FMNH 49371; Fig 5B). All premaxillary teeth are similar in size, and are arranged along the curvature of the pars dentalis of the bone. The maxillary tooth row is slightly longer than that of the premaxilla, containing some 18−20 teeth (e.g., FMNH 49371, CIB 17309, 17313). The maxillary teeth are large anteriorly, becoming shorter posteriorly towards the end of the tooth row (S4B and S4C Fig), which terminates at or close to the posterior extremity of the maxilla.

The palatal teeth occur only on the vomer, whereas the parasphenoid and pterygoid are both entirely edentate (Fig 5; S1D–S1F Fig). The number of vomerine teeth is slightly variable ontogenetically and individually. All specimens display four to six small teeth in one tooth row, with the exception of CIB 2010072723 and CIB 17308; the former shows as many as eight and the latter has no teeth on the left vomer as an abnormal condition (Fig 5C). Vomerine teeth are arranged to form a short arc, slightly bowed anteriorly. The tooth rows are located medial to the choana and are set widely apart from each other (Fig 5; S1D–S1F Fig).

The dentary tooth row, containing 22−24 teeth in each mandibular ramus, extends from the mandibular symphysis to the mid-length of the mandible, where the coronoid process of the prearticular begins to ascend (Fig 6A–6C). All dentary teeth are closely spaced, and gradually decrease in height towards the posterior end of the tooth row (S4D Fig). Following the contour of the snout, the dentary tooth row curves anteromedially to the mandibular symphysis, but more posteriorly follows a straight line to the end of the row.

### Hyobranchial apparatus

Anatomy of the hyobranchial apparatus in *Batrachuperus yenyuanensis* has not previously been documented. By micro-CT scanning and double-color staining specimens, we are able to provide a description of both cartilaginous and bony structures of the apparatus in this species.

**Fig 7:**
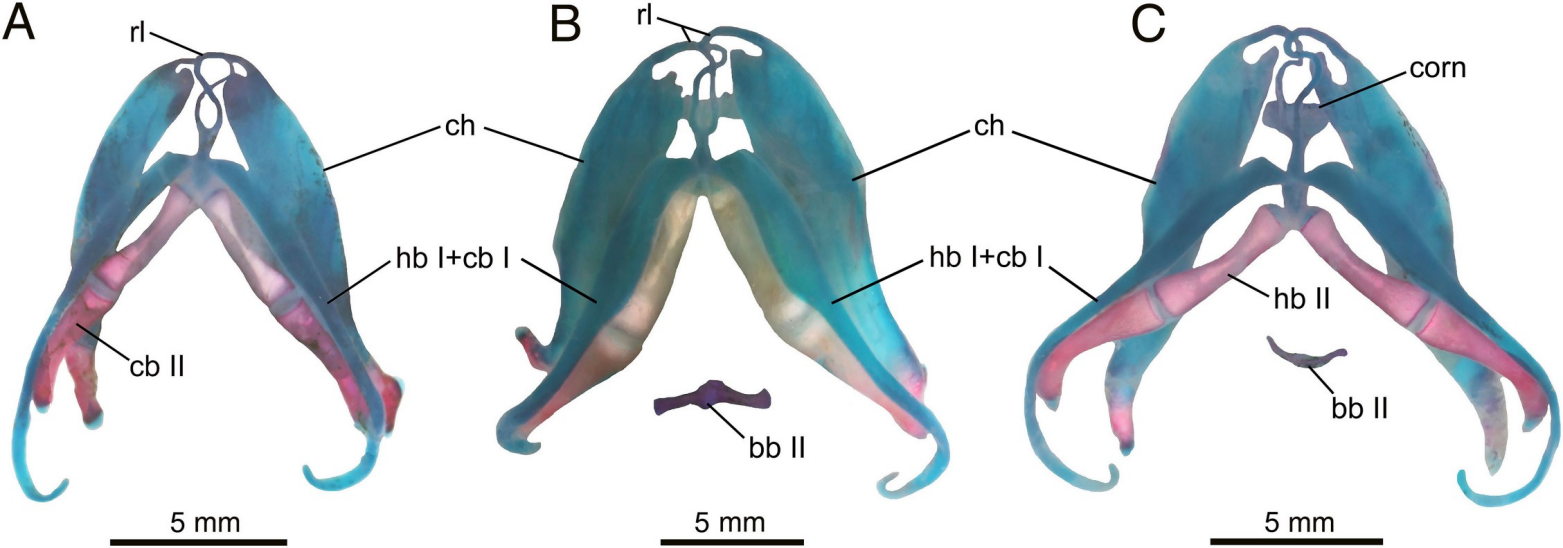
Hyobranchial apparatus of *Batrachuperus yenyuanensis* in dorsal view. A, CIB 201707YY04 (juvenile); B, CIB 201707YY09 (subadult); C, CIB 201707YY08 (adult). All three specimens from Shuangertang, Yanyuan County.

**Fig 8:**
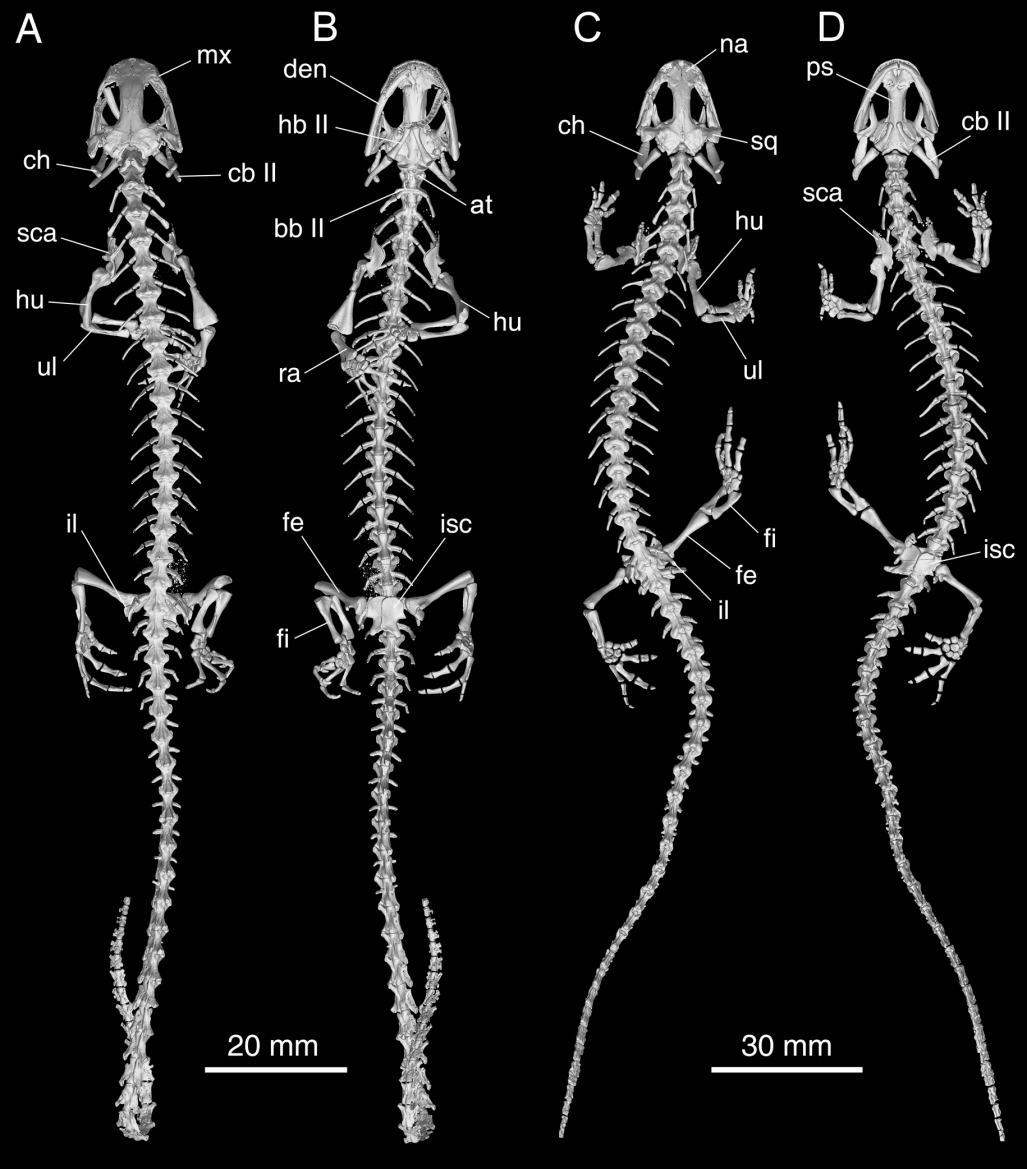
Micro-CT rendered reconstructions of the whole body skeleton of *Batrachuperus yenyuanensis*. A, B, CIB 17308 in dorsal (A) and ventral (B) views; C, D, CIB 72599 in dorsal (C) and ventral (D) views. CIB 17308 from Shuangertang, Yanyuan County; CIB 72599 from Tuowu, Mianning County.

In the median row of the apparatus, the cornua is a small cartilaginous plate, rectangular shaped and transversely positioned between the paired ceratohyals and anterior to the paired hypobranchial I (Fig 7B and 7C). Basibranchial I (anterior copula) remains cartilaginous in all specimens, with the exception of a single specimen (CIB 72593: TL = 170.13 mm) displaying partial ossification as a small knob (S1E Fig). A pair of radial loops stem from basibranchial I, extend anteriorly, then curve and cross one another to display a Fig 8-shaped pattern (Fig 7). The anterior end of the radial loop merges with the ceratohyal without any interruption. The Fig 8-shaped pattern is similar to that in *Batrachuperus pinchonii* [40], but is different from the condition in *B*. *londongensis*, in which the radial loops do not cross one another [6, 26]. Posteriorly, basibranchial II (posterior copula or os thyroideum in reference [17]) is the only ossified median element in the hyobranchium in all adult specimens. The element in most adult specimens is an inverted T-shaped structure (e.g., CIB 17305, 17308, 17309, 17314, 72595, 72597, 72599), whereas in subadults (e.g., FMNH 49371, CIB 17310, 17313, 72593, 201707YY09) and a few adults (e.g., CIB 17310, 72593, 201707YY08) basibranchial II is often retained with a small posteromedian process, rendering the element more or less cross-shaped (Fig 7; S1E and S1F Fig). This posteromedian process will eventually be resorbed as individuals mature into fully grown adults.

Among the paired lateral elements, hypobranchial I and ceratobranchial I of the first branchial arch are entirely cartilaginous and fused into a single element, as shown in cleared and stained specimens (Fig 7). The only exception known is CIB 72599, in which hypobranchial I is partly ossified. The fused condition is also known for two other species (*B*. *pinchonii* and *B*. *tibetanus*) in the same genus [6, 26, 40], but differs from that of *B*. *londongensis*, in which hypobranchial I and ceratobranchial I remain as separate elements. In *B*. *yenyuanensis*, the distal half of the first branchial arch is narrowed as a slender rod and strongly curved posteromedially to form a hook (Fig 7).

Hypobranchial II and ceratobranchial II are fully ossified as elongate rods in adults, with the latter slightly longer than the former in all specimens. The paired hypobranchial II converge anteriorly and slightly curve posterolaterally, whereas the paired ceratobranchial II curve posterodorsally (Fig 7). Ceratobranchial III and IV are entirely lost in all post-metamorphic individuals, a common pattern in metamorphic salamanders [17, 36].

The paired ceratohyals are large elements of the hyoid arch that are more dorsolaterally located than other hyobranchial elements. The element is a long and widened band, with a narrowed distal end curved posteromedially (Fig 7). The element is largely cartilaginous, but has its distal end ossified to variable extents in different specimens in keeping with their ontogenetic differences: it is ossified distally for no more than one-third of its total length in relatively small specimens (CIB 17305, 17309, 17313, 17314, 2010072723) versus one-half or over one-half the length of the element in relatively large specimens (FMNH 49371, CIB 17308, 17310, 72594–72597, 72599). Because large specimens display more extensive ossification of the ceratohyal, increased ossification of the element ontogenetically seems to be a general trend. Partial ossification of the ceratohyal is known for all other species of *Batrachuperus*, and is commonly seen in several other hynobiids as well (see reference [26] for discussion and citations).

### Axial skeleton

The vertebral column in *Batrachuperus yenyuanensis* consists of a total of 17 presacral vertebrae including the atlas, plus a single sacral, three caudosacrals, and up to 33 caudal vertebrae (e.g., CIB 17305, 17308). Out of the 37 specimens under study, 30 invariantly display 17 presacrals, with the 18^th^ vertebra bearing the single sacral vertebra (Fig 8). CIB 201707YY02 is exceptional in having 18 presacrals. Six other specimens (CIB 14548, 71309, 71313, 72594, 72597, 72599) are abnormal in having two vertebrae carrying a sacral rib on one side or the other (S5 Fig). Based on the ratios of sampled specimens, we interpret having 17 presacrals (30 of 37 specimens) as the normal pattern for *B*. *yenyuanensis*. Three other congeners (*B*. *pinchonii*, *B*. *tibetanus*, *B*. *karlschmidti*) are known to have the same number (17) of presacrals [26], whereas the remaining three congeners display variable numbers (*B*. *londongensis*: 18; *B*. *taibaiensis*: 18; *B*. *cochranae*: 16). Within Hynobiidae, *Onychodactylus fischeri* has as many as 20−22 presacrals [41], whereas other hynobiids normally have 15−16 vertebrae in the trunk series [41].

The atlas is wider anteriorly than posteriorly in both dorsal and ventral views (Fig 8; S1, S5 and S6 Figs). In lateral view, the atlas displays a large crest as a distinct feature of the neural arch, which posteriorly bears a pair of postzygapophyses in articulation with the prezygapophyses of the first trunk vertebra. Neither transverse processes nor ribs are associated with the atlas, a common condition seen in most salamanders. A robust odontoid process (tuberculum interglenoideum) projects anteroventrally from the atlas, reinforcing the cotyle-condylar articulation of the vertebral column with the skull. Lateral to the odontoid process, the atlantal cotyles are weakly concave and roughly cup-shaped, for articulation with the occipital condyles. Posterior to the cotyles, the atlas is bilaterally penetrated by a small foramen (S6A Fig), through which passes the first pair of spinal nerves, a general pattern in all urodeles [42].

The trunk vertebrae are subequal in length, with an elongate, spindle-like, and deeply amphicoelous centrum. The centrum is smooth ventrally, with no subcentral keel or subcentral foramen as in all other hynobiid species (S6B Fig). Dorsally, the neural spine displays a low ridge extending over the neural arch, the posterodorsal end of which rises as a small tubercle for ligamentous attachment of the M. interspinalis [24]. Each trunk vertebra has a pair of posterolaterally directed transverse processes in articulation with unicapitate ribs. A shallow groove is seen on both anterior and posterior surfaces of the process, indicative of fusion of the dorsal diapophysis with the ventral parapophysis. The transverse processes and the ribs associated with the first three trunk vertebrae are slightly stouter than those of other vertebrae, and their associated ribs are slightly spatulate distally for attachment of the M. thoracic-scapularis [24]. All transverse processes along the trunk series are penetrated horizontally by a foramen (S6B Fig) for passage of the arteria vertebralis [24]. No trunk vertebrae show penetration by spinal nerve foramina: the spinal nerves exit intervertebrally as in other hynobiids and cryptobranchids generally [42]. The ribs are similar in length for the first 11–12 pair, but gradually decrease in size posteriorly, with the last rib in the trunk region being merely a short triangular stub.

With the exception of the abnormal conditions in several specimens as described above, all other specimens show the normal pattern with a single sacral vertebra in articulation with a pair of sacral ribs. The sacral is similar in size to the presacrals; the transverse processes of the sacral vertebra, however, are more robust and more posterolaterally directed than those in the trunk series. The robust transverse process in the sacral is also penetrated antero-posteriorly by a foramen for passage of the arteria vertebralis (S6C Fig), as in the trunk series. The sacral rib is notably longer and stouter than any of the trunk ribs, and curves posteroventrally in a ligamentous connection with the ilium.

There are three caudosacrals, with the two anterior ones bearing no haemal arch but the third having a haemal arch that is strongly slanted posteriorly (Fig 8; S6D Fig; see reference [43] for definition of caudosacrals). The number of caudosacral vertebrae is phylogenetically significant, as plesiomorphic conditions in urodeles can have three or more caudosacrals, whereas the derived condition displays one or two [38]. The first caudal vertebra articulates with a pair of short free ribs, whereas the others have no free ribs but bear a pair of transverse processes. All but the posteriormost two or three caudals have a haemal arch attached to the ventral side of the centrum (S6E Fig). The number of caudal vertebrae varies ontogenetically, with ossification of the caudal series proceeding antero-posteriorly. Adult specimens examined by us have a maximum of 33 caudal vertebrae.

### Pectoral girdle and forelimb

**Fig 9:**
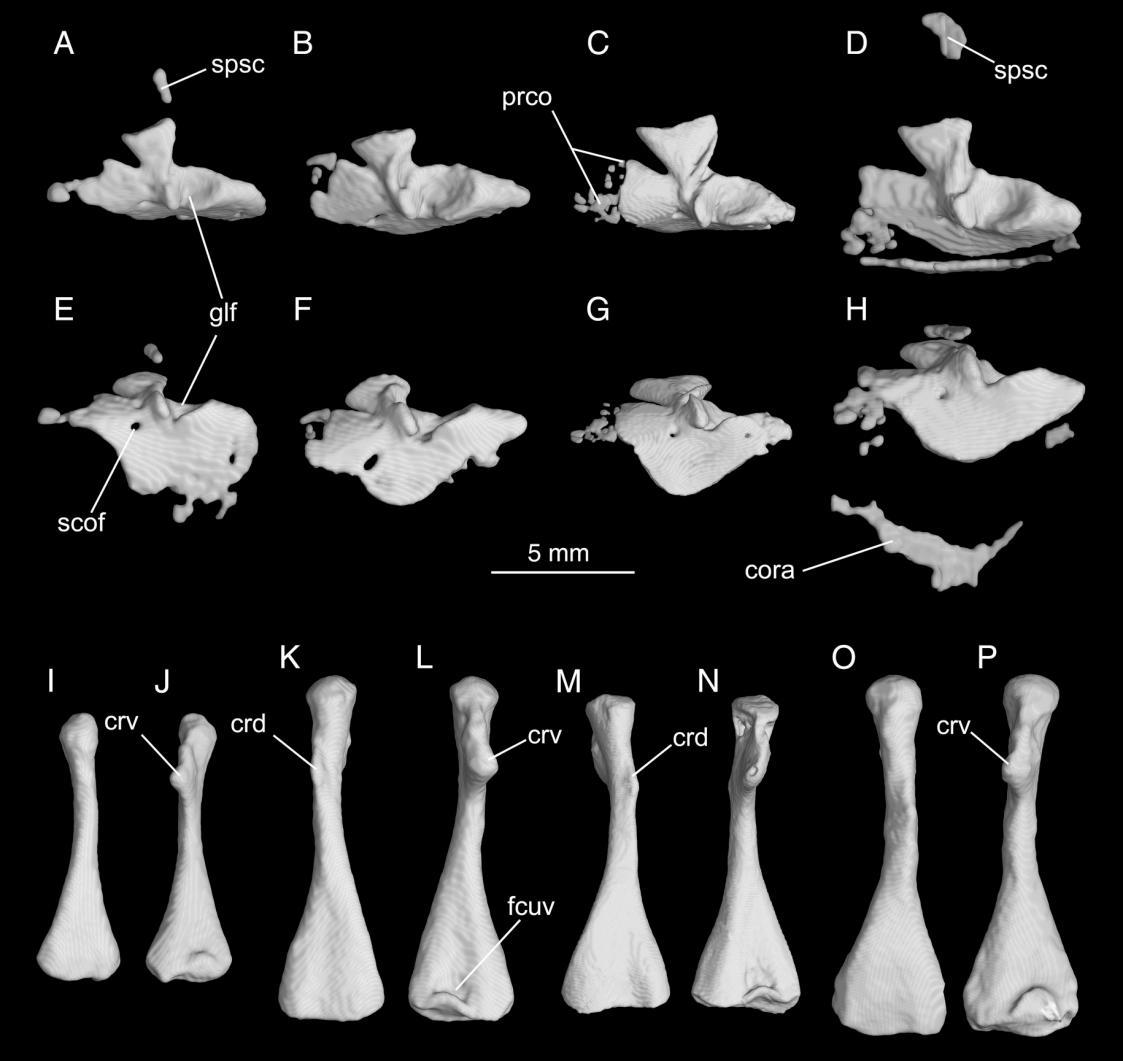
Micro-CT rendered reconstructions of the pectoral girdle and upper arm of *Batrachuperus yenyuanensis*. Left scapulocoracoid of CIB 94631 (A, E), CIB 16999 (B, F), CIB 17308 (C, G), CIB 72599 (D, H) in lateral (upper row) and ventrolateral (middle row) views; left humerus of CIB 94631 (I, J), CIB 17308 (M, N), CIB 72599 (O, P) and right humerus of CIB 16999 (K, L) in lateral (left) and medial (right) views. CIB 16999 from Shenguozhuang, Yuexi County; CIB 17308 from Shuangertang, Yanyuan County; CIB 72599 from Tuowu, Mianning County; CIB 94631 from Xieka, Jiulong County.

The scapula and coracoid are co-ossified into a single scapulocoracoid unit as in most other salamanders, except for sirenids, which display separate scapula and coracoid [44]. As a morphological feature shared with other congeneric species, the scapular blade in *Batrachuperus yenyuanensis* is extremely short, even shorter than the height of the coracoid plate (Fig 9A–9H). The blade shows a slightly widened dorsal border, but is more constricted at its junction with the coracoid plate.

Posteroventral to the scapular blade, the glenoid fossa is a large depression that receives the humeral head. On the anterior rim of the fossa is a prominent bony buttress that functions in reinforcing articulation of the limb (humeral head) with the girdle (glenoid fossa). Between the buttress and the lower rim of the glenoid fossa is a deep incision (Fig 9A–9H) that receives the ventral crest of the humerus when the arm swings forward. The coracoid plate is expanded both antero-posteriorly and ventrally, and is slightly curved medially. The ventral border of the plate is grooved for articulation with the cartilaginous part of the coracoid. A large foramen supracoracoideum penetrates the coracoid plate anteroventral to the glenoid fossa, serving for passage of the nervus supracoracoideus and the associated vessels [24]. In addition to the co-ossified scapulocoracoid, extra-ossification of normally the cartilaginous suprascapular, procoracoid and coracoid regions is observed in several specimens (Fig 9A–9H).

The humerus is straight and fairly robust, with a short and narrow shaft, but strongly expanded proximal and distal ends (Fig 9I–9P). Proximally, the humeral head merges with a large and triangular ventral crest (crista ventralis humeri), onto which the MM. pectoralis and supracoracoideus attach [24]. Also at the proximal end, a short trochlear groove on the flexor side furnishes a space receiving the bony buttress described above. On the extensor side, the dorsal crest (crista dorsalis humeri) is a short, but prominent ridge extending along the post-axial border to the humeral shaft (Fig 9I, 9K, 9M and 9O). As observed in several specimens (e.g., CIB 17308, 72594, 72595, 72599), the dorsal crest is often marked with a small, but prominent tubercle for insertion of the M. subscapularis as in other salamanders [24]. Distally, the ulnar and radial condyles are well ossified in adults, allowing a bone-to-bone articulation with their corresponding elements in the forearm. The radial condyle is slightly larger than the ulnar condyle; ventrally between the condyles, a deep fossa cubitalis ventralis receives the proximal end of the radius when the forearm is flexed.

**Fig 10:**
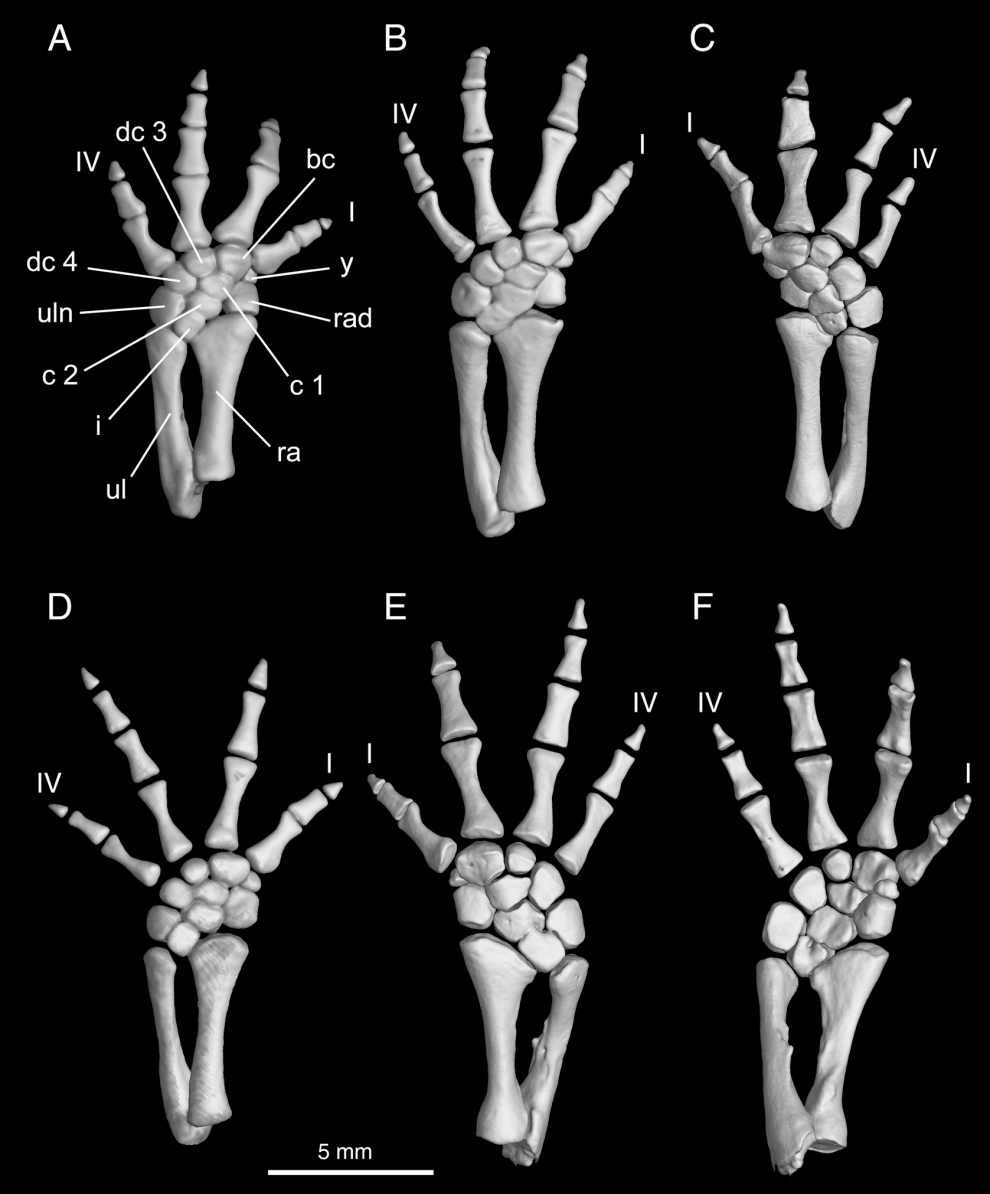
Micro-CT rendered reconstructions of the forearm and manus of *Batrachuperus yenyuanensis*. Left forearm and manus of CIB 94631(A), CIB 17313 (B), CIB 72596 (D) in lateral view; right forearm and manus of FMNH 49371 (C) and CIB 17308 (E) in dorsal and lateral views; right forearm and manus of CIB 17308 in ventral and medial views (F). FMNH 49371, CIB 17308 and CIB 17313 from Shuangertang, Yanyuan County; CIB 72596 from Tuowu, Mianning County; CIB 94631 from Xieka, Jiulong County.

In the forearm, the ulna and radius are roughly equal in length (Fig 10). The radius is essentially straight, whereas the ulna bows slightly laterally, with its proximal end bending toward the radius. Close to its proximal end and on the posterior aspect, the radius bears a prominent tubercle (e.g., CIB 17308) that marks the ligamentous insertion of the elbow flexor M. humero-antibrachialis [24]. The ulna is more strongly expanded at its proximal than its distal end, with an oblique and slightly concave surface for attachment of the cartilaginous ulnar process; conversely, the radius is wider at its distal than proximal end. Posterolaterally, the ulna bears a crest as the insertion of the M. extensor antibrachii ulnaris (CIB 17308).

Maximum ossification of the mesopodium of the forelimb includes a total of nine elements (e.g., FMNH 49371, CIB 14550, 17302, 17305, 17308, 17313, 17314, 72593, 72596, 72597; Fig 10). The intermedium is relatively small and is penetrated by a foramen, for passage of the arteria perforans carpi [24]. The triangular proximal portion of the intermedium is wedged between the radius and ulna; distally the intermedium contacts the proximal centrale, and posteriorly it contacts the ulnare. It is worth noting that *Batrachuperus yenyuanensis* is the only species within the genus that retains two centralia in the manus, whereas all other species have only one centrale [26]. Although fusion of the proximal and distal centralia does occur as individual variations in some sampled specimens, possibility of ontogenetic fusion of the elements can be ruled out because it is seen in both small (e.g., CIB 17002, TL = 133.82 mm) and large (CIB 72599, TL = 231.39 mm) individuals. Random fusion of other carpal elements is also observed in several specimens, including: fusion of intermedium with ulnare in CIB 72592, 72594; fusion of basale commune with element y and radiale in CIB 14514. It also should be noted that the proximal centrale in *B*. *yenyuanensis* is in direct contact with the radius, although in a limited extent; the direct contact of the two elements prevents the intermedium from meeting the radiale (Fig 10).

As a pre-axial element, the radiale is a large bone, roughly the same size as either of the two centralia. Distal to the radiale is a small element y that articulates distally with metacarpal I. In the distal row, the basal commune is the largest carpal bone, roughly twice the size of the element y, and distally contacts both metacarpal I and II. Distal carpal 3 is slightly larger than element y, and the former is set between the distal centrale and metacarpal III. More laterally, distal carpal 4 can be twice the size of distal carpal 3, and it articulates with the centralia and ulnare.

Four digits are developed in the manus, a common pattern in urodeles. Digit 3 is the longest, followed in decreasing lengths by digits 2, 4 and 1 (Fig 10). No consistent patterns are evident for the digital formula in the manus: the paratype FMNH 49371 displays 2-2-2-1 on the right but 2-2-x-2 on the left (digit 3 is missing); 2-2-2-2 on both sides in CIB 17305, whereas other specimens display a mixture of 2-2-2-1 and 2-2-3-2 in CIB 17308, 2-1-2-2 and 1-2-2-1 in CIB 17309, 2-2-3-2 and 2-2-2-1 in CIB 17310, 2-2-2-2 and 2-2-3-2 in CIB 17314. Despite the variable conditions of the digital formula in the forefoot, the sequence of relative lengths of the digits as we described seems to be consistent.

### Pelvic girdle and hindlimb

**Fig 11:**
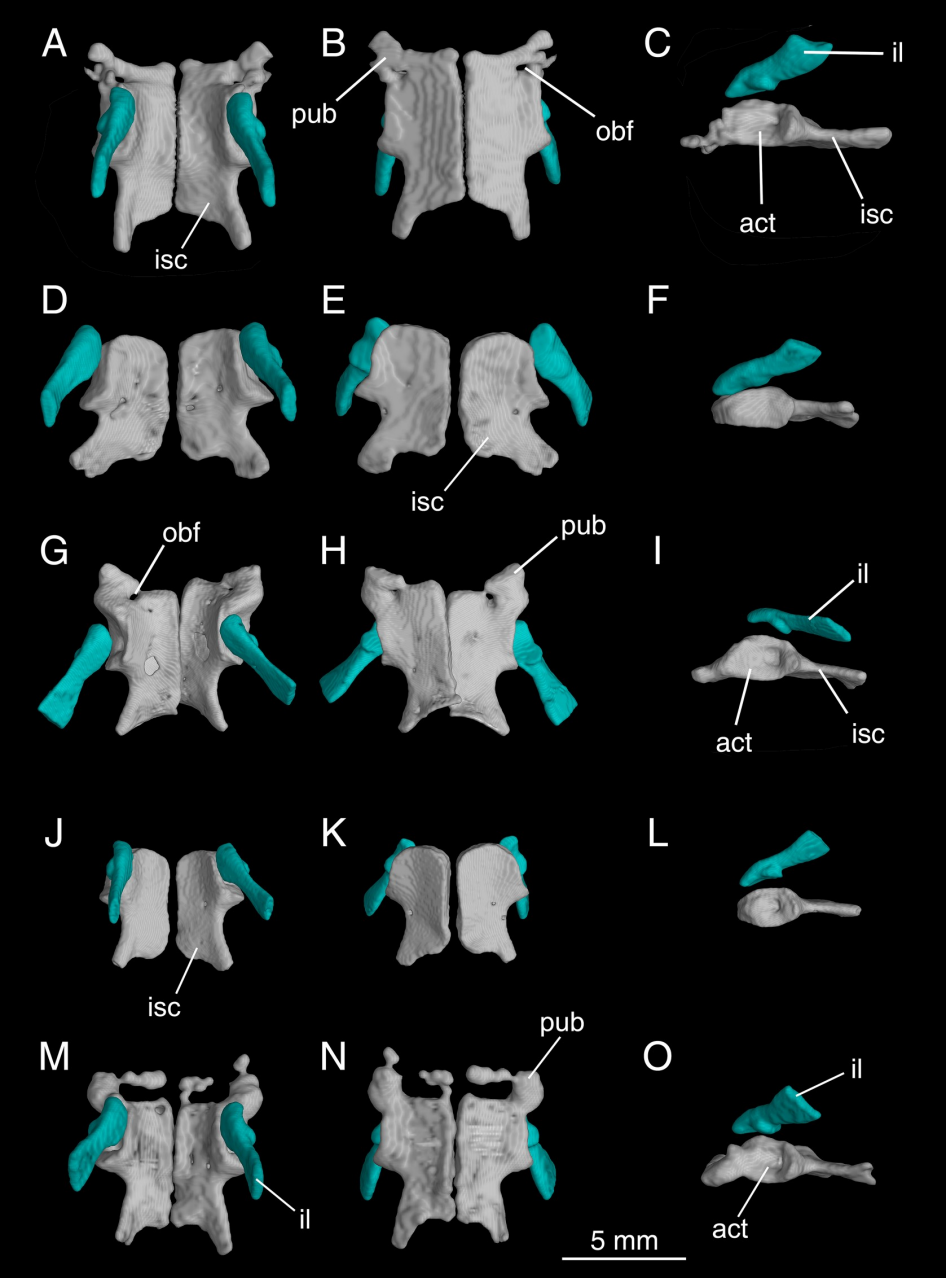
Micro-CT rendered reconstructions of the pelvic girdle of *Batrachuperus yenyuanensis* in dorsal (left column), ventral (middle column) and left lateral (right column) views. CIB 14514 (A–C), CIB 16999 (D–F), CIB 17302 (G–I), CIB 17307 (J–L) and CIB 72592 (M–O). Note partly ossified pubis in CIB 14514 (A–C), CIB 17302 (G–I), and CIB 72592 (M–O). CIB 14514 from Maoniudui, Dechang County; CIB 16999 from Shenguozhuang, Yuexi County; CIB 17302 and CIB 17307 from Shuangertang, Yanyuan County; CIB 72592 from Tuowu, Mianning County.

**Fig 12:**
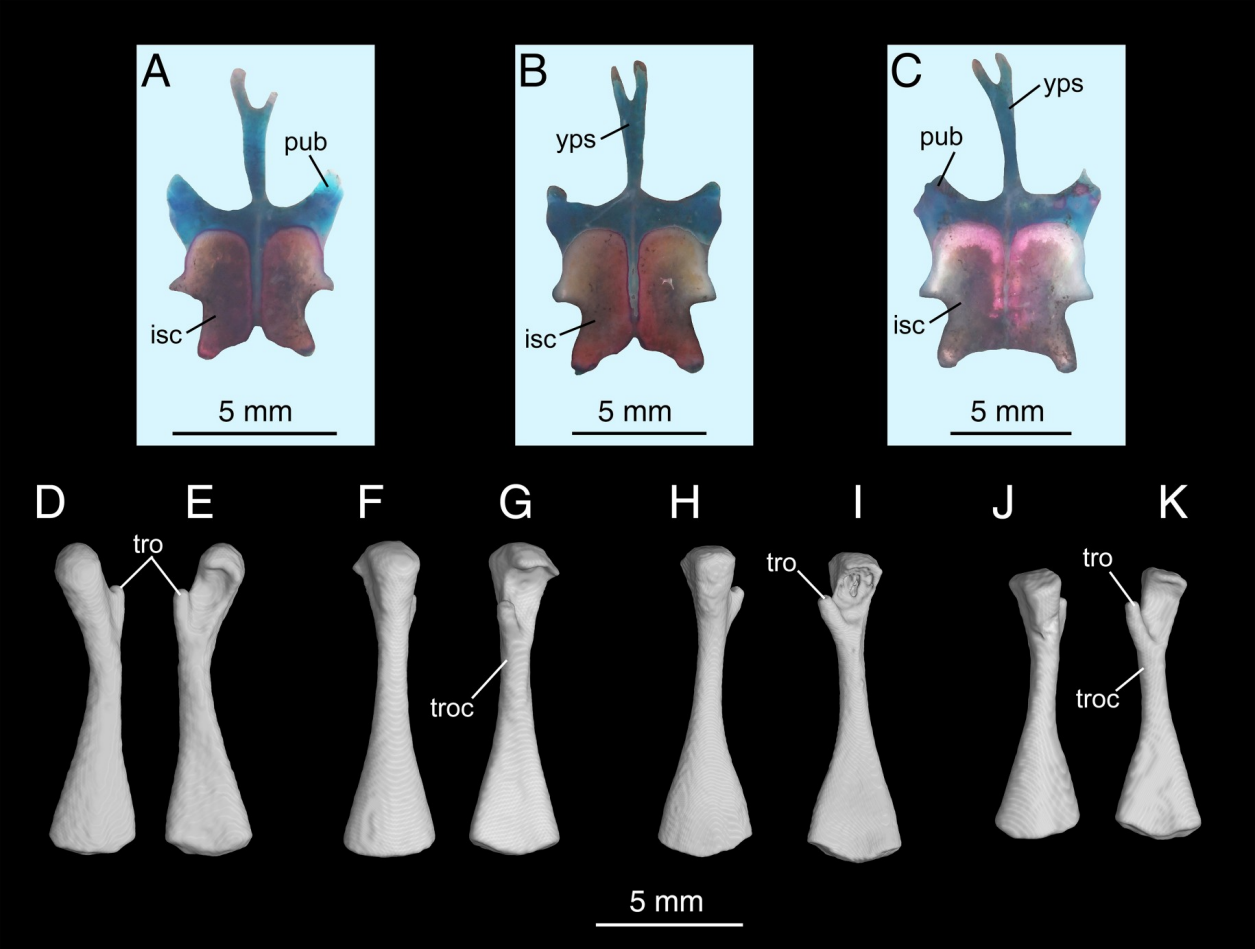
Micro-CT rendered reconstructions of the ypsiloid cartilage and femur of *Batrachuperus yenyuanensis*. Ypsiloid cartilage in dorsal view in CIB 201707YY04 (A), CIB 201707YY08 (B), CIB 201707YY09 (C); right femur of CIB 14514 (D, E), CIB 16999 (F, G), CIB 17308 (H, I) and CIB 94632 (J, K) in dorsal (left) and ventral (right) views. CIB 14514 from Maoniudui, Dechang County; CIB 16999 from Shenguozhuang, Yuexi County; CIB 17308, 201707YY04, 201707YY08 and 201707YY09 from Shuangertang, Yanyuan County; CIB 94632 from Xieka, Jiulong County.

The pelvis (Fig 11) displays the morphology seen in most other salamanders, having the paired ilia and ischia fully ossified, but with the pubes remaining cartilaginous (but see below). The ypsiloid as a median element is entirely cartilaginous as documented in cleared-and-stained specimens (Fig 12A–12C).

The ilium is roughly club-shaped, consisting of a narrow blade projecting posterodorsally from the acetabulum and a slightly expanded ventral plate that is irregular in shape and contributes to most of the dorsal part of the acetabulum (Fig 11). The iliac blade has a slightly constricted neck immediately above the acetabulum. The acetabulum is a large fossa that receives the femoral head. The dorsal rim of the acetabulum is a prominent buttress arising from the ilium, whereas the ventral rim is entirely from the ischium with no involvement of the pubis even though partial ossification of the latter indeed occurs in some specimens (see below).

The ischium is a large plate, roughly quadrangular shaped in ventral view. The paired ischial plates meet along the midline to form a hinged articulation at the symphysis (Fig 11). In those specimens lacking partial ossification of the pubis, each plate has a rounded anterior border, the rim of which is grooved for articulation with the cartilaginous pubis (Fig 11D–11F and 11J–11L). This rounded border merges laterally with a triangular ischial process at the posteroventral border of the acetabulum. This process locks into the trochlear groove of the femur when the hindlimb swings backward. Immediately posterior to the ischial process, the posterior part of the ischial plate shows a notched lateral border, followed by a short and robust ischial spine onto which attaches the M. ischio-caudalis, a flexor of the tail [24]. Many specimens show perforation of the ischium by a foramen (e.g., CIB 17005, 17307, 17310, 17313, 17314), as has been documented in *B*. *londongensis* [26].

The pubis remains cartilaginous in some specimens, but partly ossified in the others as seen in *Batrachuperus londongensis* [26]. Partial ossification of the pubis is commonly seen in large or relatively large specimens examined for *B*. *yenyuanensis* (e.g., CIB 14514, 14548, 14550, 17302, 17310, 72592–72597, 72599, 92592–92596, 92599). Resulting from this extra ossification in the pelvis, the obturator foramen can be observed as penetrating the pubo-ischium plate or partially open at the anterior border of the ischium (Fig 11A–11C and 11J–11L).

A Y-shaped ypsiloid is present as a slender cartilage as part of the pelvis, as in other species of the same genus and in all hynobiids as well. This cartilaginous element is not shown in micro-CT scanned images, but is observed in all of the three cleared and stained specimens (Fig 12A–12C). The ypsiloid stems from the cartilaginous pubes, extends anteriorly and terminates with a bifurcated end below the 16^th^ presacral vertebra.

The femur is roughly the same length as, but is more robustly built than, the humerus. The proximal end of the femur is expanded, bearing a large femoral head in all adults. Along the post-axial border of the femur the dorsal trochanteric crest arises as a straight bony ridge, extending distally from the femoral head to the fibular condyle (Fig 12D, 12F, 12H and 12G). This bony ridge marks the insertion of the M. pubo-ischio-femoralis internus, a powerful extensor of the upper leg in salamanders [24]. Proximally on the flexor side, the femoral trochanter projects to form a robust and hooked process, onto which inserts the M. pubo-ischio-femoralis externus as in other salamanders [45]. This process is separated from the femoral head by a large, U-shaped notch (Fig 12E, 12G, 12I and 12K). The ventral trochanteric crest is a much weaker ridge, and extends from the femoral trochanter distally to the tibial condyle. The trochlear groove is a deep trough between the dorsal and ventral trochanteric crests. At the distal end, the tibial and fibular condyles are fully ossified, enabling a bone-to-bone articulation with their corresponding elements in the lower leg (see below).

**Fig 13:**
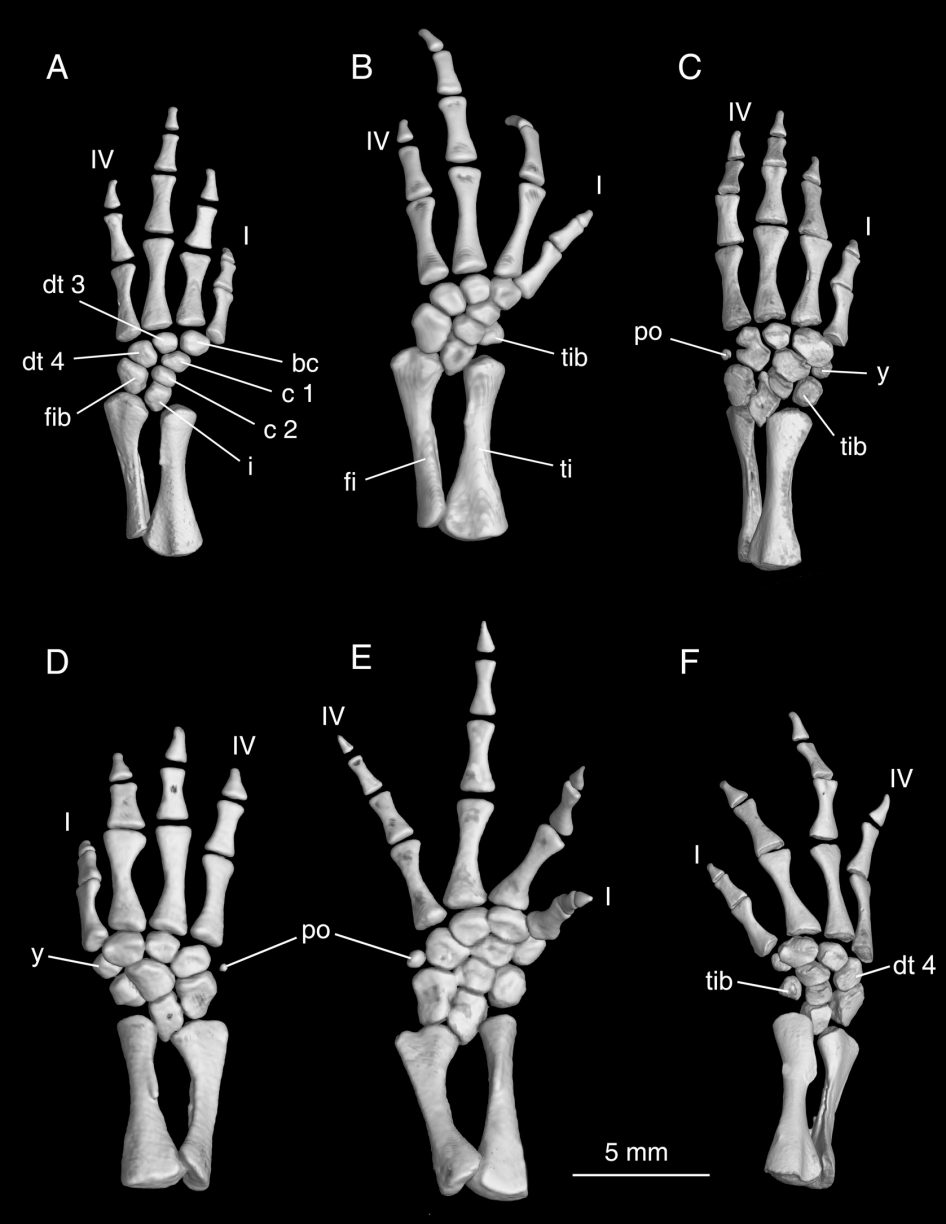
Micro-CT rendered reconstructions of the lower hindlimb and pes of *Batrachuperus yenyuanensis*. Left lower hindlimb of CIB 17309 (A), CIB 17313 (B), FMNH 49371 (C) and CIB 72597 (E); right lower hindlimb of CIB 14514 (D) and FMNH 49371 (F). Note the preaxial elements (tibiale and/or element y) are delayed in ossification in CIB 17309 and CIB17313; the two centralia are fused in CIB 14514 but remain separated from each other in all other specimens; the postminimus is ossified in FMNH 49371, CIB 14514 and CIB 72597. CIB 14514 from Maoniudui, Dechang County; CIB 17309, 17313, and FMNH 49371 from Shuangertang, Yanyuan County; CIB 72597 from Tuowu, Mianning County.

Both the tibia and fibula are short elements, each roughly similar in length (Fig 13). The tibia has an expanded proximal end, with a prominent tibial crest arising from the extensor surface for a ligamentous connection with the femur. Distally along the medial side, the tibia bears a small crest for insertion of the M. extensor cruris tibialis [45]. In contrast to the tibia, the fibula is more expanded distally than proximally. It has a concave medial border, but is laterally straight (Fig 13).

Maximum ossification of the mesopodium in the hindlimb includes as many as ten tarsal elements (Fig 13C and 13E). Among the three basal elements, the intermedium is roughly diamond-shaped, wedged between the tibia and fibula proximally and between a centrale and the fibulare distally. As in the forelimb, the intermedium is perforated by a foramen for passage of the arteria perforans tarsi [24]. The tibiale is rounded, slightly smaller than the intermedium. It is medially in contact with the two centralia, and distally articulates with an even smaller element y (Fig 13C–13E). The fibulare, slightly larger than the intermedium, is in articulation with the intermedium, proximal centrale and distal tarsal 4. As in the forelimb, two centralia are present in the pes in many specimens (e.g., FMNH 49371, CIB 14550, 17302, 17305, 17308, 17313, 72592–72596, 94631, 94632). By contrast all other species of *Batrachuperus* have only one centrale element. Distally, element y is slightly smaller than the tibiale, whereas the basale commune is a large element in contact with both metatarsals I and II. Distal tarsal 3 and 4 are roughly the same size, articulating with their corresponding metatarsals. A bony postminimus is absent in many specimens, but is ossified as a small knob in several large and presumably fully-grown adults (e.g., CIB 72592, 72597, 72599), thus, indicating delayed ossification of this element as has been documented in *B*. *londongensis* [26].

There are four digits in the pes, a derived pattern in mountain stream salamanders in comparison with the generalized five digit pattern in most hynobiids. Metatarsal II and III are subequal in length, whereas IV is significantly longer than I. In terms of relative length of the digits, digit 3 is the longest, digits 2 and 4 are subequal, and digit 1 is the shortest (Fig 13). The same pattern is also seen in all other species of the genus [6]. As in the forelimbs described above, the paratype FMNH 49371 also displays an asymmetrical development of the hindlimbs: the left pes has a digital formula of 2-2-3-3, but 2-2-3-2 on the right. Comparison of all available specimens shows no consistent pattern for the species: two specimens (CIB 17314, 2010072723) display a digital formula of 2-2-3-2 on both sides, another two specimens (CIB 17305, 17309) show 2-2-2-2 on both sides; whereas the remainder display a mixture of 2-2-2-2 and 2-2-3-2 on left/right or right/left sides.

## Discussion

### Osteological characterization of *Batrachuperus yenyuanensis*

Resulting from this study, three features listed in previous publications as diagnostic for the species are now recognized as ontogenetic and/or individual variations; and thus, have been removed from our revised diagnosis of the species. We discuss these characters below.

The lacrimal entering the external naris anteriorly, but not the orbit posteriorly, has been regarded as diagnostic for *Batrachuperus yenyuanensis* [6, 8, 26]. However, our examination of a large number of specimens from different localities shows that the shape and sutural patterns of the lacrimal in relation to its surrounding elements are both ontogenetically and individually variable at post-metamorphic stages. Generally with ontogeny, the shape of the lacrimal changes from more or less triangular to L-shaped in dorsal view. That the lacrimal enters only the external naris can be indeed observed in many specimens we examined, but these are mostly subadults as indicated by limited ossification of the articular in the mandibles and incomplete ossification of the mesopodia in the limbs. On the other hand, several specimens (e.g., CIB 16999, 17005, 72599) show that the lacrimal anteriorly enters the border of the external narial opening and also posteriorly enters the rim of the orbit. Interestingly, these include not only large and presumably fully-grown adult specimens (TL = > 200 mm) having fully ossified articulars and mesopodia (e.g., CIB 72599), but also juveniles (e.g., CIB 94627; TL = 132 mm) having unossified articulars and limited ossification of the mesopodia, and medium-sized subadults having incompletely ossified articulars and mesopodial elements (e.g., CIB 16999: TL = 181 mm; CIB 17005: TL = 183 mm). The evidence seems to indicate that patterns of the lacrimal in relation to the narial and orbital openings are highly variable ontogenetically and individually in *B*. *yenyuanensis*. Therefore, we have removed the lacrimal entering only the naris from our revised diagnosis of the species.

The second putative diagnostic feature concerns the size of the anterodorsal fenestra between narial openings. The anterodorsal fenestra is described as a large opening in *Batrachuperus yenyuanensis* (premaxillary fontanel in reference [8]) without quantified values. Our study of a large number of *B*. *yenyuanensis* specimens allows us to recognize two different patterns: many medium-sized specimens with a TL of ~140–180 mm (e.g., CIB 17002, 17309) display the fenestra as roughly one-half the size of the external narial opening, whereas large specimens with a TL of ~180–230 mm (e.g., CIB 17308, 72599) tend to show a much smaller fenestra being no more than one-third the size of the naris. Reduction of the size of the fenestra seems to result from more extensive ossification of the nasal bone. In contrast to the lacrimal condition discussed above, the size of the anterodorsal fenestra in relation to the narial opening seems to correspond well with body size: small to medium-sized specimens (TL = ~90–180 mm) with less extensive ossification of the nasals tend to have a large fenestra, roughly two-thirds the size of the narial opening; in contrast, large specimens (TL = ~180–230 mm) tend to have a proportionally small fenestra, no more than one-third the size of the narial opening. We quantified the size of the anterodorsal fenestra in adults as one-half to one-third of the external narial opening as being diagnostic for the species.

The third feature concerns the presence of a pillar-like bony structure resulting from perichondral ossification of the ascending process of the palatoquadrate. As described above (see palate and braincase description), some specimens examined in our study display a pillar-like bony rod that forms a brace supporting the lateral margin of the parietal above the otic process of the pterygoid, whereas other specimens lack such a pillar in the same position. In our recent paper on the osteology of *Batrachuperus londongensis* [26], we documented ontogenetic ossification of the pillar-like structure in the congeneric species *B*. *londongensis*, *B*. *tibetanus* and *B*. *taibaiensis*. Here, our extensive survey of specimens revealed that ontogenetic ossification of this pillar-like structure also occurs in *B*. *yenyuanensis*. For example, a large adult (CIB 72599: TL = 231 mm) from the Tuowu locality in Mianning area displays a fully ossified pillar bracing the lateral edge of the parietal against the pterygoid (Figs 3A and 4A; S2A, S2B and S3A Figs). Several other specimens from the same locality (e.g., CIB 72594: TL = 195 mm, CIB 72595: TL = 197 mm, CIB 72597: TL = 196 mm) show incomplete ossification of the pillar, a large part of which is probably still cartilaginous (S7A, S7C and S7D Fig). Interestingly, no specimens from the type locality Shuangertang show ossification of this element. CIB 17308 (TL = 201 mm) is a large adult from the type locality, but shows no sign of ossification of the element. On the contrary, CIB 17003 (TL = 144.6 mm) is a much smaller adult from the Shenguozhuang locality, but has the pillar well ossified at such a body size (S7B Fig). Comparison of the two specimens indicates that the latter is likely older by age, based on more extensive ossification of the articular in this smaller individual than the former. Nonetheless, it is now known that the ossification of the pillar is delayed after maturity, and thus using the presence/absence of the bony pillar as a diagnostic feature of a particular species must be based on extensive survey of specimens to avoid possible mis-characterization of the taxon understudy. Because of the ontogenetically variable conditions, we here recommend not using the presence or absence of the bony pillar as a diagnostic feature for species in the genus *Batrachuperus*.

Also in this study, we added several features to our diagnosis for the species based on comparison with other species in the same genus. These include: otic process of the squamosal well developed and the presence of a single mental foramen in the dentary. In the postcranial skeleton, most specimens used in this study from different localities consistently show that the vertebral column has 17 presacrals, with the exception of a single specimen (CIB 201707YY02) having 18 presacrals. This feature can be regarded as diagnostic for *B*. *yenyuanensis* in combination with other features. Although having 17 presacrals is not unique but shared with several other species of the same genus (*B*. *pinchonii*, *B*. *tibetanus*, *B*. *karlschmidti*), it differs from presacral counts of 16 and 18 in other congeners.

### Confirmation of geographic occurrences of the species outside Yanyuan area

**Fig 14:**
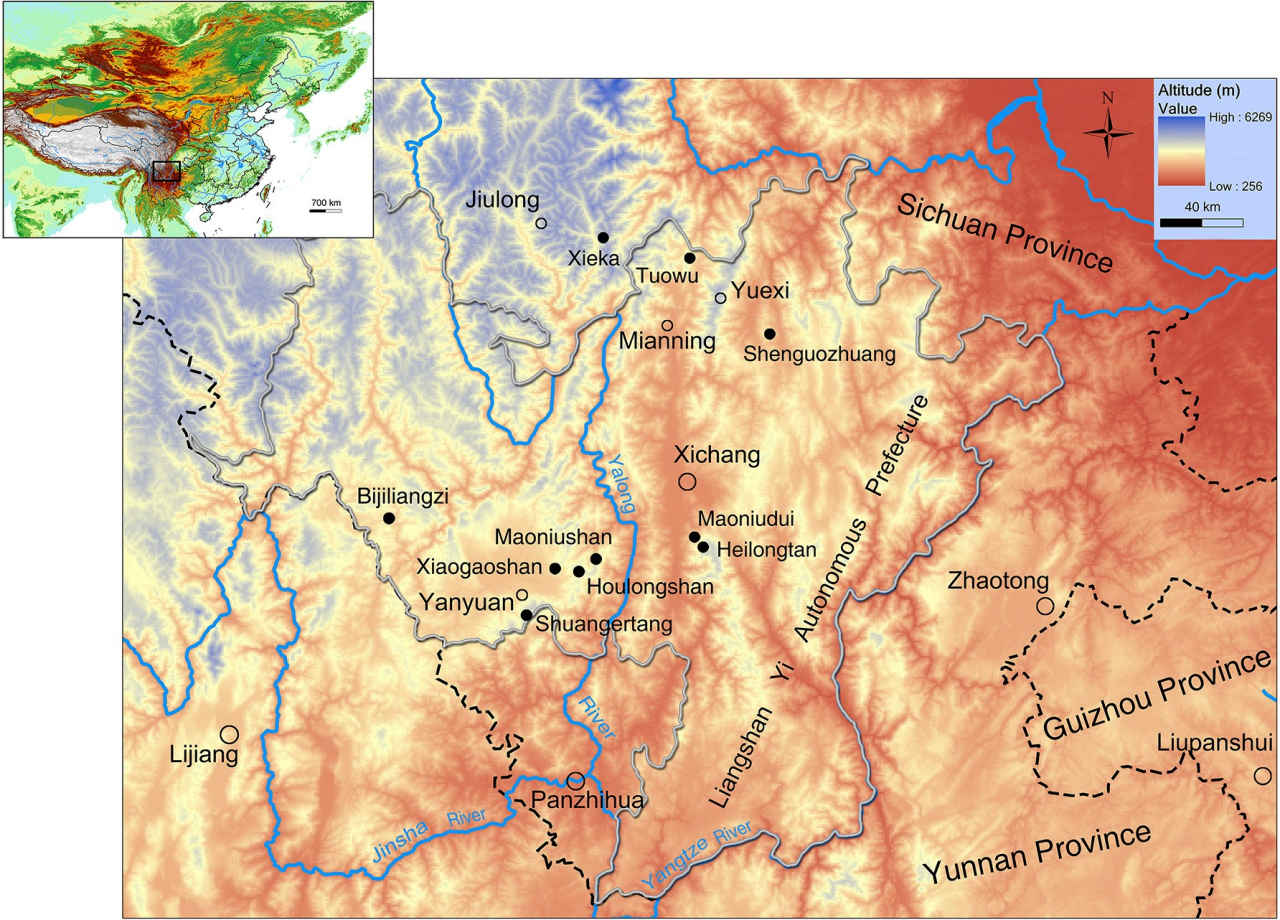
Patterns of geographic distribution of *Batrachuperus yenyuanensis* in western Sichuan Province of China. Note that localities (solid dots) are spread on both the west and east side of the Yalong River (see S1 Table for altitude information of the localities). Small circles denote county towns and large circles denote city towns in the study area.

Besides the type locality (Shuangertang) at Bailinshan, occurrences of *Batrachuperus yenyuanensis* have been recorded from several other localities in the Yanyuan area [46]: i.e., Xiaogaoshan (meaning “Little Tall Hill”; ~20 km northeast of Shuangertang), Maoniushan (meaning “Yak Hill”; ~40 km northeast of Shuangertang), Houlongshan (~28 km northeast of Shuangertang), and Bijiliangzi (meaning “Biji Crest”: ~60 km northwest of the county town of Yanyuan). These localities have an elevation range of 2900−3700 m above sea level [46] and all are geographically clustered west of the Yalong River (Fig 14).

Outside the type Yanyuan area, possible occurrences of *Batrachuperus yenyuanensis* have been reported from several localities in the Xichang and Mianning areas, but these occurrences have been treated as questionable for the last decade [6, 8, 11]. In the Xichang area, the two localities are Heilongtan (meaning “Black Dragon Pool”; N27°58′176′′, E102°37′972′′, ~35 km southeast of Xichang) and Maoniudui (meaning “Yak Brigade”; N102°32′502′′, E27°60′995′′, ~30 km southeast of Xichang). For our study, we sampled and micro-CT scanned a total of seven specimens from these two localities (see S1 and S2 Tables).

Northwards of Xichang, in the northern part of Liangshan Yi Autonomous Prefecture, *Batrachuperus yenyuanensis* has been reported from two localities. The Tuowu locality is approximately 30 km north of the county town of Mianning (Fig 14). From this locality, the species has been recorded from the same mountain stream at elevations of 3600 m (sub-locality I: N28°49′564′′, E102°16′594′′) and 2440 m (sub-locality II: N28°57′028′′, E102°17′784′′) above sea level [7]. At sub-locality II, the lowest elevation range (2440 m) is recorded for the species. We sampled a total of eight specimens from the Tuowu locality, including both young and fully grown adults, with TL ranging from 170−231 mm. The Shenguozhuang locality (N28°32′002′′, E102°45′659′′; altitude 2856 m above sea level; ~17 km southeast of township Puxiong) is approximately 37 km southeast of the county town of Yuexi (Fig 14). We sampled four specimens from this locality.

Outside of the Liangshan Yi Autonomous Prefecture, *Batrachuperus yenyuanensis* has been reported from the Xieka locality (N28°55′646′′, E101°50′336′′, altitude 3116 m above sea level), approximately 33 km southeast of the county town of Jiulong and ~45 km northwest of Tuowu, in Jiulong County, Ganzi Tibetan Autonomous Prefecture [7]. We sampled three specimens from the Xieka locality, a post-metamorphic juvenile (CIB 94627) and two subadults (CIB 94631, 94632).

We micro-CT scanned sample specimens from all of the above-listed localities (S1 and S2 Tables) and made osteological comparisons of them based on 3D reconstruction of the CT-scanning images. Comparison of the specimens from these localities with those from the type locality Shuangertang confirm that they all pertain to the same species, namely *Batrachuperus yenyuanensis*: they all display the same body plan with a flat skull slightly longer than wide, and a tail evidently longer than the SVL with a tail fin as a thin crest. Other diagnostic features of the species observed in these specimens from different localities include: anterodorsal fenestra roughly one-third to one-half the size of the external naris; vomerine teeth four to six in number, arranged in a short arc that bows anteriorly; a single mental foramina opens within the anterior one-quarter below the dentary tooth row; presacral vertebrae 17 in number; and retention of two centralia in both fore- and hind limbs. Confirmation of occurrences of the species in these areas along the Yalong River provides information for understanding patterns of geographic distribution of the species in western Sichuan Province, along the east fringe of the Qinghai-Tibetan Plateau (see discussion below).

### Patterns of distribution and historical biogeography of *Batrachuperus yenyuanensis*

As has been mentioned above, *Batrachuperus yenyuanensis* has a confined geographic distribution in western Sichuan Province. All occurrences of the species are known within the territory of the Liangshan Yi Autonomous Prefecture, plus a single occurrence at the Xieka locality in Ganzi Tibetan Autonomous Prefecture in the same province (Fig 14). A close look at the distribution map reveals several notable features. First, all the geographic occurrences of the species are within a narrow zone, extending ~180 km north−south along the east fringe of the Qinghai-Tibetan Plateau (Fig 14). Second, all the occurrences are along a section of the Yalong River, a major tributary of the upper stream Yangtze River and associated with the Hengduan Mountains along the eastern fringe of the Qinghai-Tibetan Plateau. Third, the type locality Shuangertang, along with several other localities (Xiaogaoshan, Houlongshan, Maoniushan, Bijiliangzi) near the county town of Yanyuan, is located on the west side of the Yalong River, whereas the remaining localities are on the east side of the river (Fig 14). The patterns of the geographic distribution for *B*. *yenyuanensis* are evidence that the biogeographic origin and dispersal history of the species in this area must be closely associated with the orogeny of the Hengduan Mountains, which, in turn is closely associated with the uplift of the Qinghai-Tibetan Plateau, and the formation of the river system in the Hengduan Mountains in general, and the Yalong River in particular.

*Batrachuperus yenyuanensis* inhabits high-altitude mountain stream and pond environments at elevations ranging from 2440−4025 m above sea level [6–8]. The salamander reaches its highest elevation range of 4025 m above sea level at the type locality Shuangertang in Yanyuan area [6, 8], and its lowest range of 2440 m above sea level at the Tuowu locality near Mianning [7]. Between Mianning in the north and Yanyuan in the south, the lower stream of the Yalong River runs through the deep mountain valley associated with the Jinping Mountains. The width of the river stream ranges between 60−150 m, with the watercourse running at a velocity of 1560 m^3^/second. Such a large river with a swift watercourse in deep gorges between elevated mountain ranges (average altitude of >3500 m above sea level) would form major geographic barriers for dispersal of the salamander. Obviously, the distribution patterns of the species raise questions on when and how the salamander crossed the swift watercourse and the mountain ranges to shape up its current distribution. Molecular studies estimate 15−25 Ma for the origin of *Batrachuperus* [14]. Given the basal position of *Batrachuperus yenyuanensis* in relation to other congeneric species, the initial dispersal of the species across the Yalong River must be close to the time of origin for the genus. The time window of 15−25 Ma corresponds to the early-middle Miocene orogeny (TS_3_), during which the Hengduan Mountains began to arise along the eastern fringe of the Qinghai-Tibetan Plateau [47]. This implies an early expansion of *B*. *yenyuanensis* in western Sichuan Province before the geographic barriers were in effect, as the Qinghai-Tibetan Plateau attained its mean elevation no earlier than late Miocene (~8 Ma [47]) or as late as late Pliocene (~3.5 Ma [48]). Nevertheless, our biogeographic hypothesis about *Batrachuperus yenyuanensis* deserves to be tested by a comparison of genetic distance with other species of the same genus and also a comparison of populations of *B*. *yenyuanensis* on opposite sides of the Yalong River.

## Conclusions

Our study of bony anatomy of the *Batrachuperus yenyuanensis*, the Yenyuan Stream Salamander, comes to the following conclusions:

1. *Batrachuperus yenyuanensis* can be clearly distinguished from all other congeneric species by a suite of morphological features, most prominently: Distinct longitudinal skin folds present ventrally in the intermandibular area; anterodorsal fenestra small, roughly one-half to one-third of the size of narial opening in adults; vomerine teeth, four to six in number in each tooth row, arranged as two short arcs, bowed anteriorly and widely separated from one another; a single mental foramen opens within anterior one-fourth of dentary; and presence of two centralia in fore- and hind limb.
2. Three features previously identified as diagnostic for *Batrachuperus yenyuanensis* are recognized as individually and/or ontogenetically variable; two of these have been removed and one has been modified in our revised diagnosis of the species.
3. *Batrachuperus yenyuanensis* has a restricted geographic distribution along the eastern fringe of the Qinghai-Tibetan Plateau. All known occurrences of the species are confined within a narrow zone that extends 180 km in north-south direction, along the western and eastern sides of the Yalong River in western Sichuan Province. The current geographic distribution indicates that the origin and biogeographic history of the species are closely associated with the rise of the Hengduan Mountains and the formation of the Yalong River along the eastern fringe of the Qinghai-Tibetan Plateau.
4. Because *Batrachuperus yenyuanensis* occupies a basal position in relation to other species in the same genus, an early expansion of the species distribution in western Sichuan Province is expected. The species may well have spread across the mountain ranges and Yalong River in early-middle Miocene, and shaped up its current distribution before the geographic barriers were in effect in Pliocene.

## Supporting information

S1 Fig **Micro-CT rendered reconstruction of the skull of *Batrachuperus yenyuanensis* in dorsal (A–C) and palatal (D–F) views, showing variations of the lacrimal (red) and basibranchial II (blue)**: A, D, CIB 94627 (TL = 132.1 mm); B, E, CIB 72593 (TL = 170.13 mm); C, F, CIB 16999 (TL = 181.16 mm). Note the lacrimal enters both the naris and orbit in CIB 94627 and CIB 16999, but enters the naris only in CIB 72593; note also partial ossification of basibranchial I as a small knob in CIB 72593; the basibranchial II is inverted T-shaped in CIB 94627, cross-shaped in CIB 72593, and roughly a transverse bar in CIB 16999. CIB 16999 from Shenguozhuang, Yuexi County; CIB 72593 from Tuowu, Mianning County; CIB 94627 from Xieka, Jiulong County.
(TIF)

S2 Fig Micro-CT rendered reconstruction of the skull of *Batrachuperus yenyuanensis* in right lateral view. A, B, CIB 72599 from Tuowu locality; C, D, FMNH 49371 from Shuangertang locality; E, F, CIB 17308 from Shuangertang locality. Note the squamosal is removed in the left column to show details of the quadrate, and both squamosal and quadrate are removed in the right column to show the lateral view of the braincase.
(TIF)

S3 Fig Micro-CT rendered reconstruction of the skull and palate of *Batrachuperus yenyuanensis* with skull roof partly removed to show dorsal view of the braincase. A, CIB 72599 from Tuowu locality; B, FMNH 49371 from Shuangertang, Yanyuan County.
(TIF)

S4 Fig Marginal teeth of *Batrachuperus yenyuanensis*. A, premaxillary teeth in palatal view; B, C, maxillary teeth in labial and lingual views; D, dentary teeth in labial view. All from CIB 201707YY04 (not to scale), a subadult from the type locality Shuangertang. Dark arrows point to the dividing zone, white arrows pointing to resorption pits in (A) and pointing to replacement teeth in (C).
(TIF)

S5 Fig Micro-CT rendered reconstructions of the whole skeleton of *Batrachuperus yenyuanensis* in dorsal and ventral views. A, B, CIB 14548 from Maoniudui, Xichang; C, D, CIB 17309 from Shuangertang, Yanyuan. Note the presacral vertebrae are 16 in number in CIB 14548 but 18 in CIB 17309; note also abnormal articulation of sacral ribs with the pelvic girdle in these two specimens.
(TIF)

S6 Fig Micro-CT rendered reconstructions of vertebrae of *Batrachuperus yenyuanensis*. A, atlas; B, 8th trunk vertebra; C, sacral vertebra; D, last caudosacral vertebra; E, 9th caudal vertebra. All vertebrae from the paratype specimen (FMNH 49371) and are arranged from left to right in the following order: anterior, posterior, dorsal, ventral and left lateral views. Abbreviations: aco, anterior cotyle; dips, dipapophysis; hc, horizontal canal; hpy, haemapophysis; nar, neural arch; ncr, neural crest; opr, odontoid process; popr, postzygapophyseal process; prpr, prezygapophyseal process; prps, parapophysis; spfo, spinal foramen.
(TIF)

S7 Fig Micro-CT rendered reconstructions of the skull of *Batrachuperus yenyuanensis* in left lateral view, showing variable extent of ossification of a pillar (color coded in blue). A, CIB 17002; B, CIB 17003; C, CIB 72594; D, CIB 72595. CIB 17002, and 17003 from Shenguozhuang, Yuexi County; CIB 72594, and 72595 from Tuowu, Mianning County.
(TIF)

S1 Table Information on specimens used in this study. (DOCX)

S2 Table Parameters of micro-CT scanning of specimens used in this study. (DOCX)
