# Supplementary figures and images for: Osteology of Batrachuperus yenyuanensis (Urodela, Hynobiidae), a high-altitude mountain stream salamander from western China
Source: PLoS One. 2019 Jan 25;14(1):e0211069. doi: 10.1371/journal.pone.0211069 (PMC6347256; doi:10.1371/journal.pone.0211069)

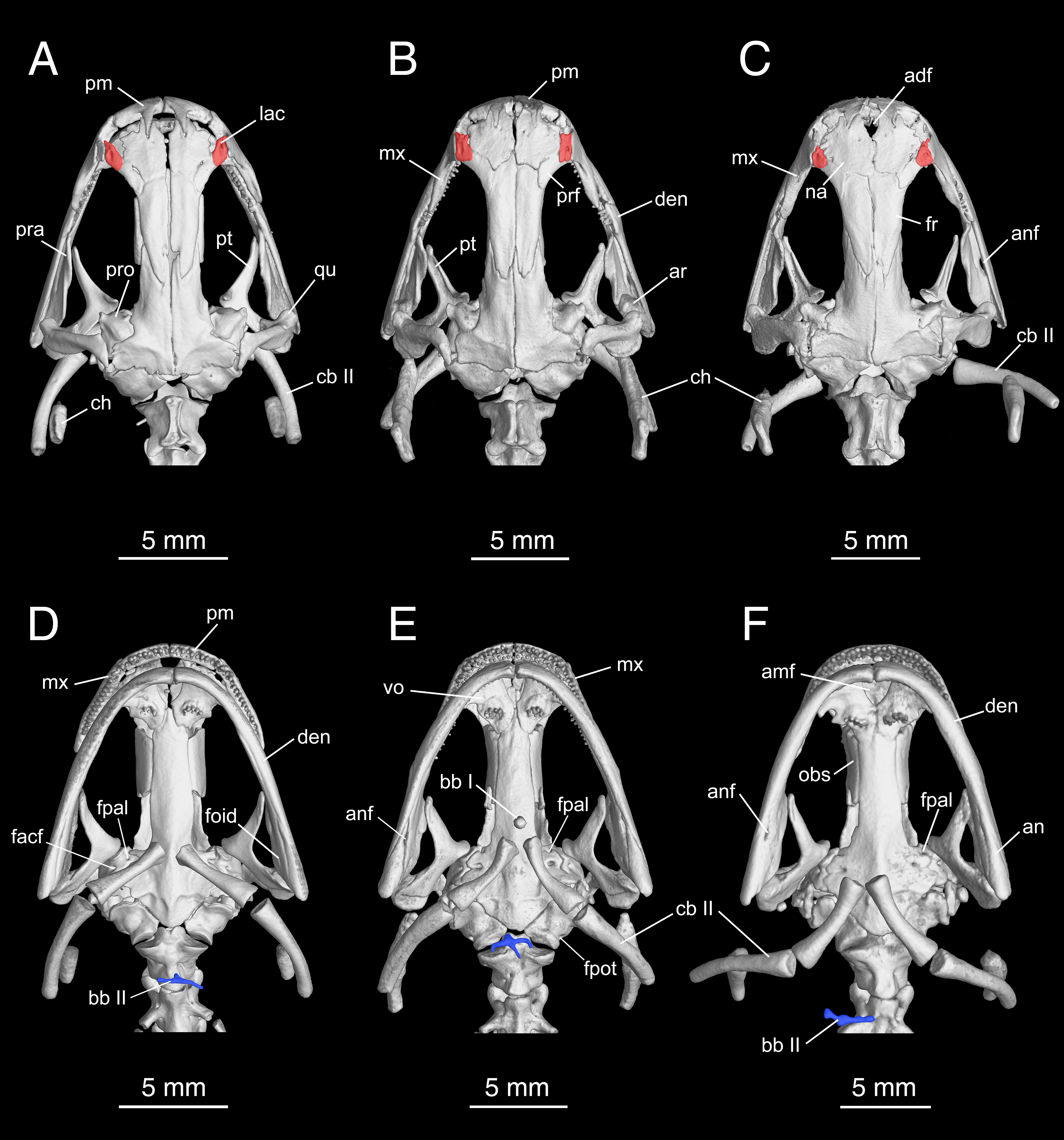

Supplement: S1 Fig — Micro-CT rendered reconstruction of the skull of Batrachuperus yenyuanensis in dorsal (A–C) and palatal (D–F) views, showing variations of the lacrimal (red) and basibranchial II (blue): A, D, CIB 94627 (TL = 132.1 mm); B, E, CIB 72593 (TL = 170.13 mm); C, F, CIB 16999 (TL = 181.16 mm). Note the lacrimal enters both the naris and orbit in CIB 94627 and CIB 16999, but enters the naris only in CIB 72593; note also partial ossification of basibranchial I as a small knob in CIB 72593; the basibranchial II is inverted T-shaped in CIB 94627, cross-shaped in CIB 72593, and roughly a transverse bar in CIB 16999. CIB 16999 from Shenguozhuang, Yuexi County; CIB 72593 from Tuowu, Mianning County; CIB 94627 from Xieka, Jiulong County. (TIF) [file pone.0211069.s001.tif]

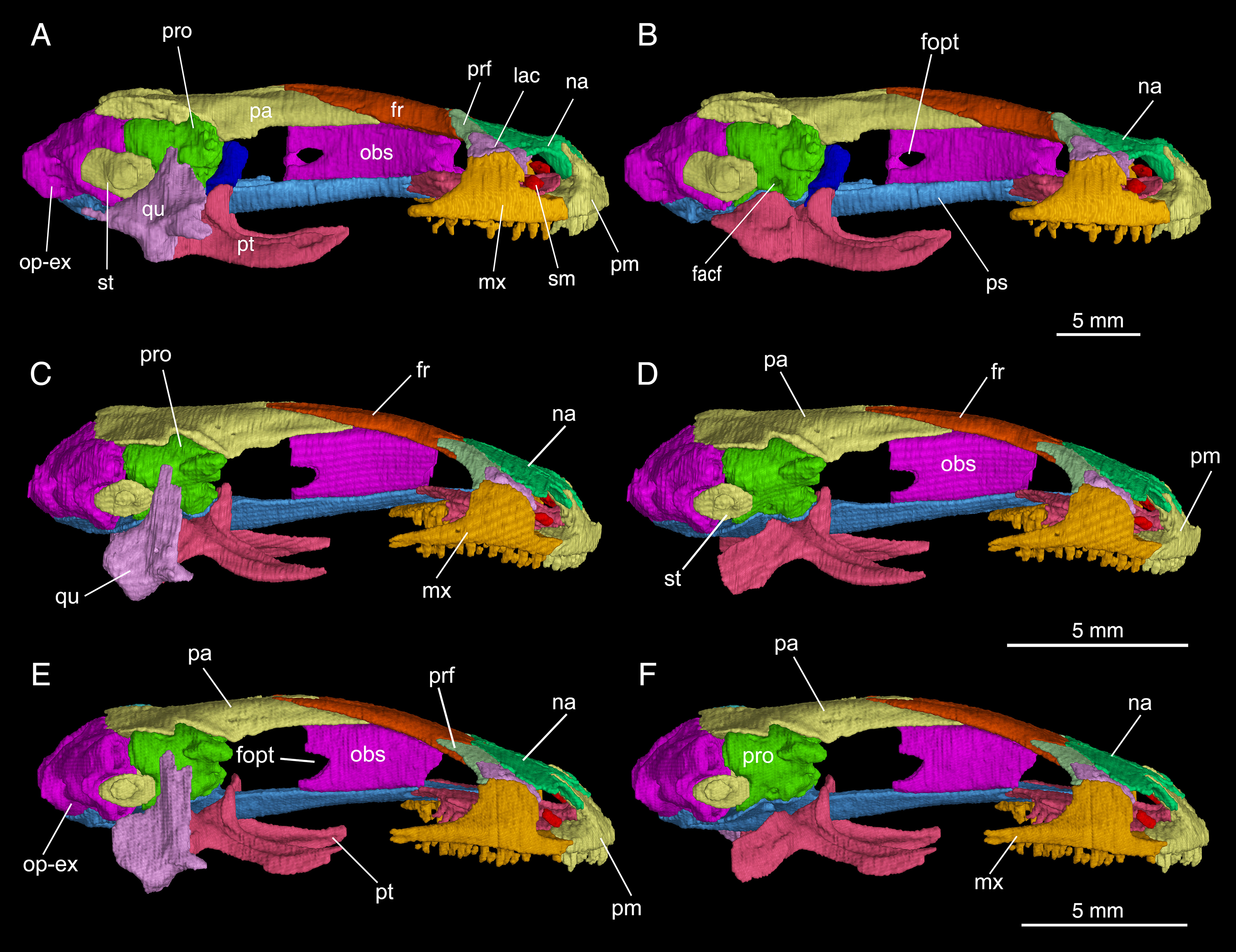

Supplement: S2 Fig — A, B, CIB 72599 from Tuowu locality; C, D, FMNH 49371 from Shuangertang locality; E, F, CIB 17308 from Shuangertang locality. Note the squamosal is removed in the left column to show details of the quadrate, and both squamosal and quadrate are removed in the right column to show the lateral view of the braincase. (TIF) [file pone.0211069.s002.tif]

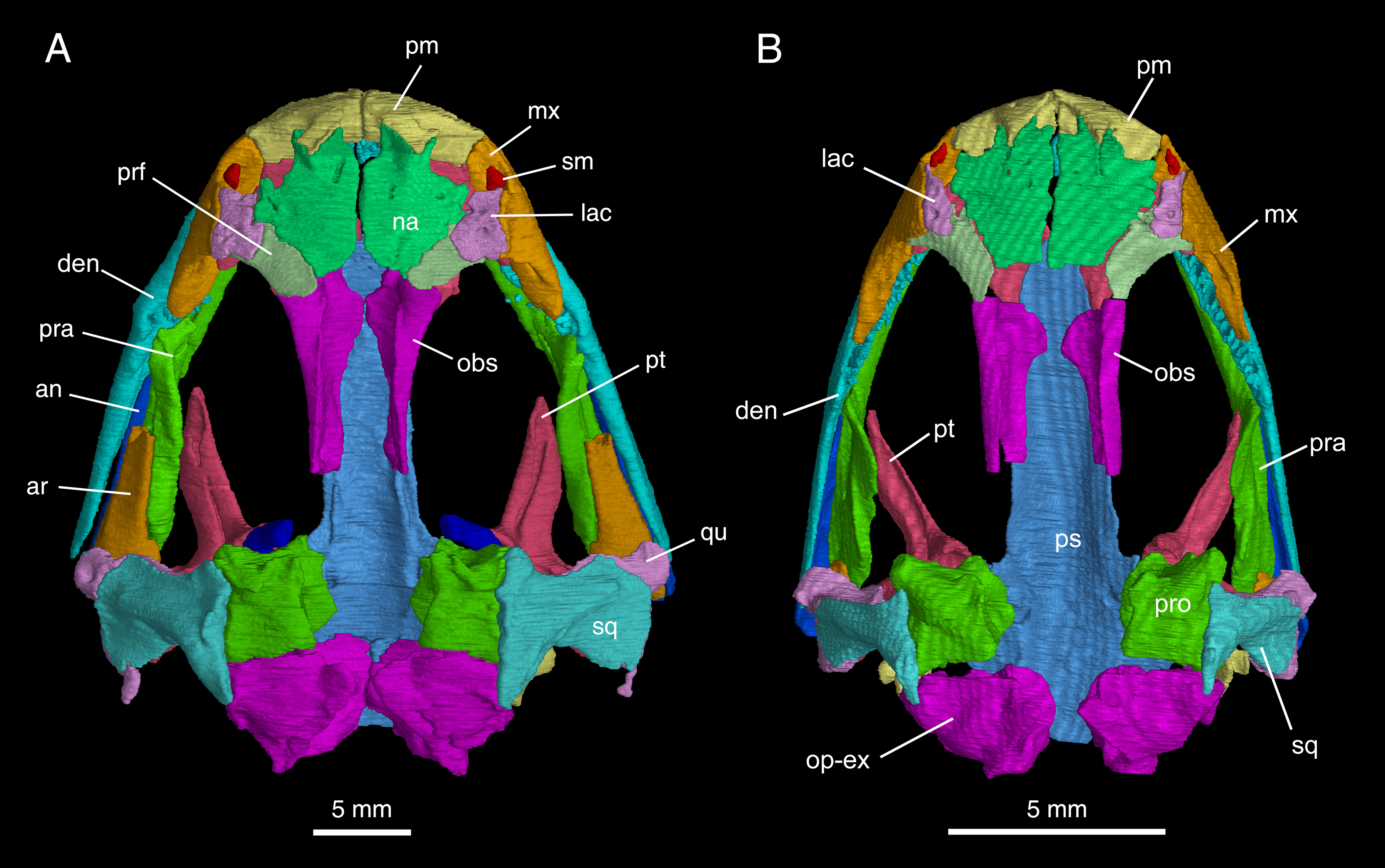

Supplement: S3 Fig — A, CIB 72599 from Tuowu locality; B, FMNH 49371 from Shuangertang, Yanyuan County. (TIF) [file pone.0211069.s003.tif]

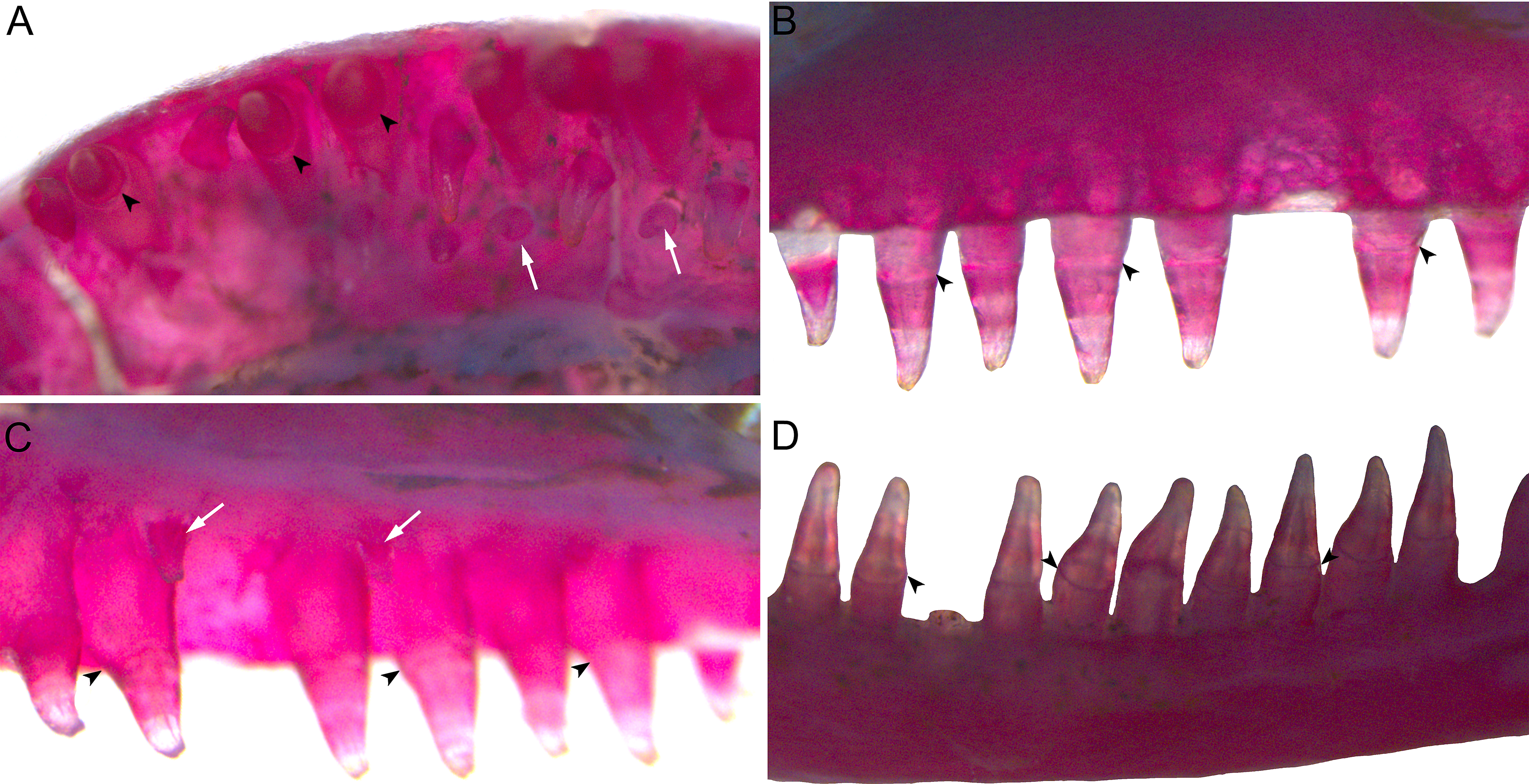

Supplement: S4 Fig — A, premaxillary teeth in palatal view; B, C, maxillary teeth in labial and lingual views; D, dentary teeth in labial view. All from CIB 201707YY04 (not to scale), a subadult from the type locality Shuangertang. Dark arrows point to the dividing zone, white arrows pointing to resorption pits in (A) and pointing to replacement teeth in (C). (TIF) [file pone.0211069.s004.tif]

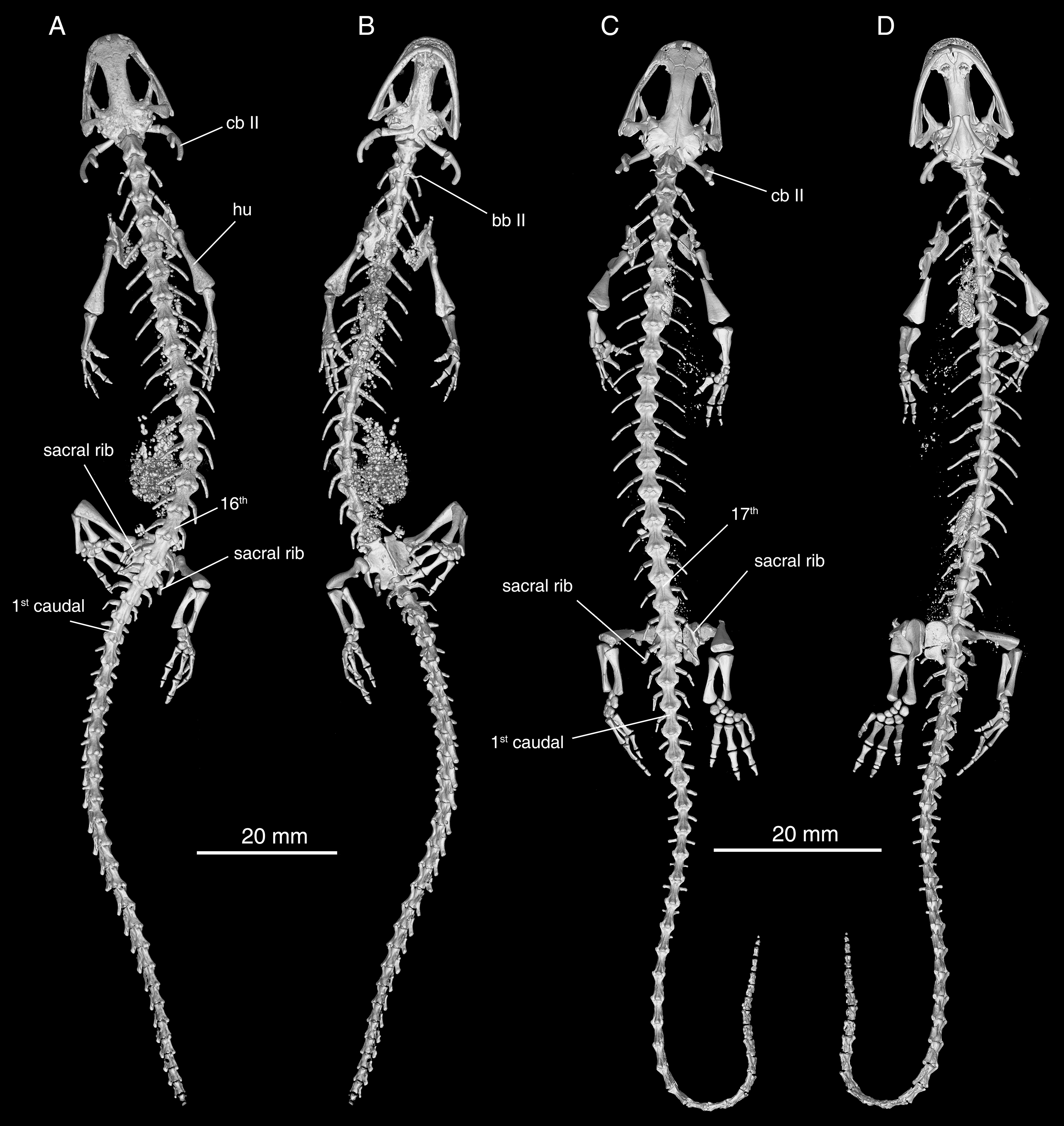

Supplement: S5 Fig — A, B, CIB 14548 from Maoniudui, Xichang; C, D, CIB 17309 from Shuangertang, Yanyuan. Note the presacral vertebrae are 16 in number in CIB 14548 but 18 in CIB 17309; note also abnormal articulation of sacral ribs with the pelvic girdle in these two specimens. (TIF) [file pone.0211069.s005.tif]

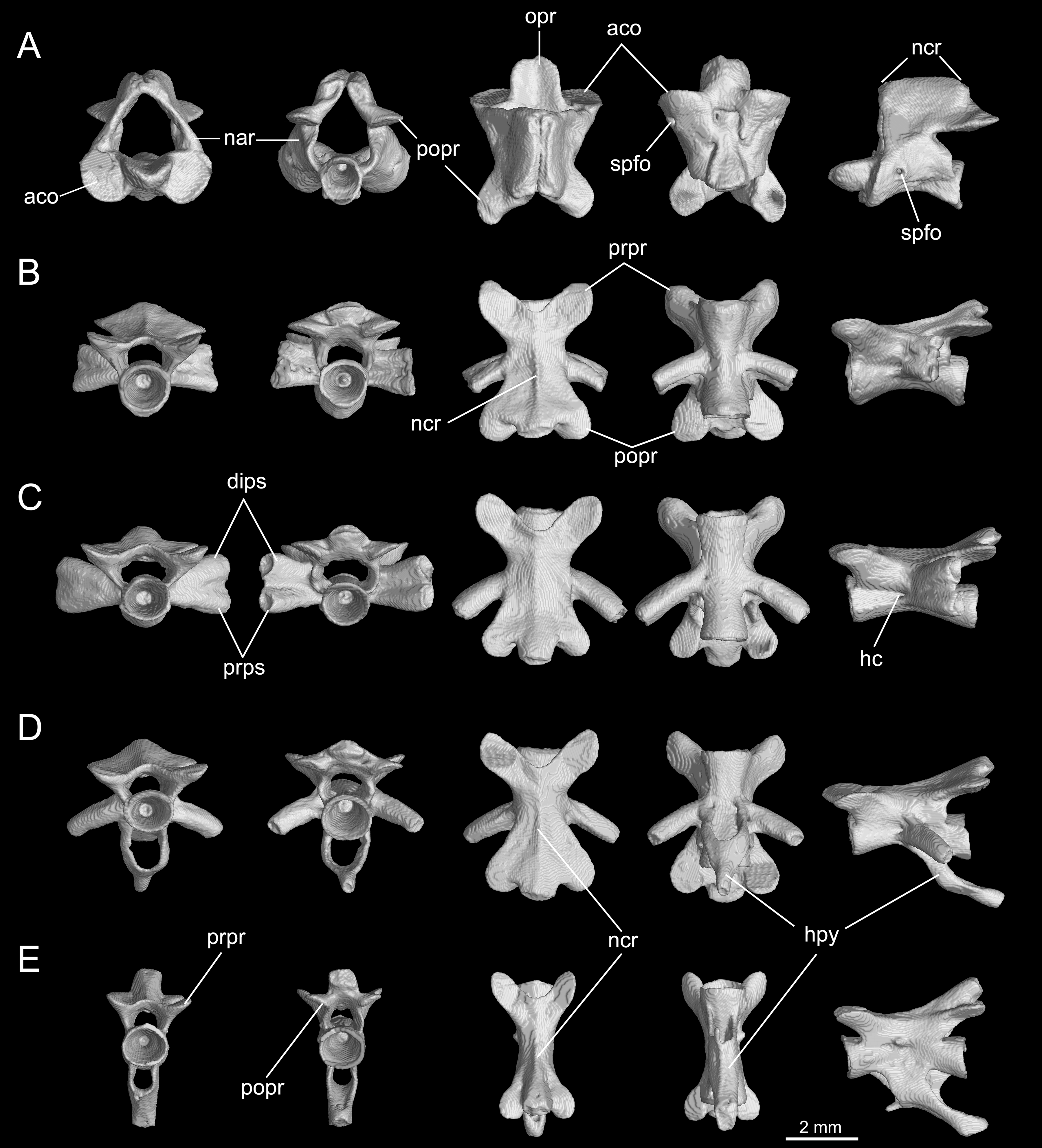

Supplement: S6 Fig — A, atlas; B, 8th trunk vertebra; C, sacral vertebra; D, last caudosacral vertebra; E, 9th caudal vertebra. All vertebrae from the paratype specimen (FMNH 49371) and are arranged from left to right in the following order: anterior, posterior, dorsal, ventral and left lateral views. Abbreviations: aco, anterior cotyle; dips, dipapophysis; hc, horizontal canal; hpy, haemapophysis; nar, neural arch; ncr, neural crest; opr, odontoid process; popr, postzygapophyseal process; prpr, prezygapophyseal process; prps, parapophysis; spfo, spinal foramen. (TIF) [file pone.0211069.s006.tif]

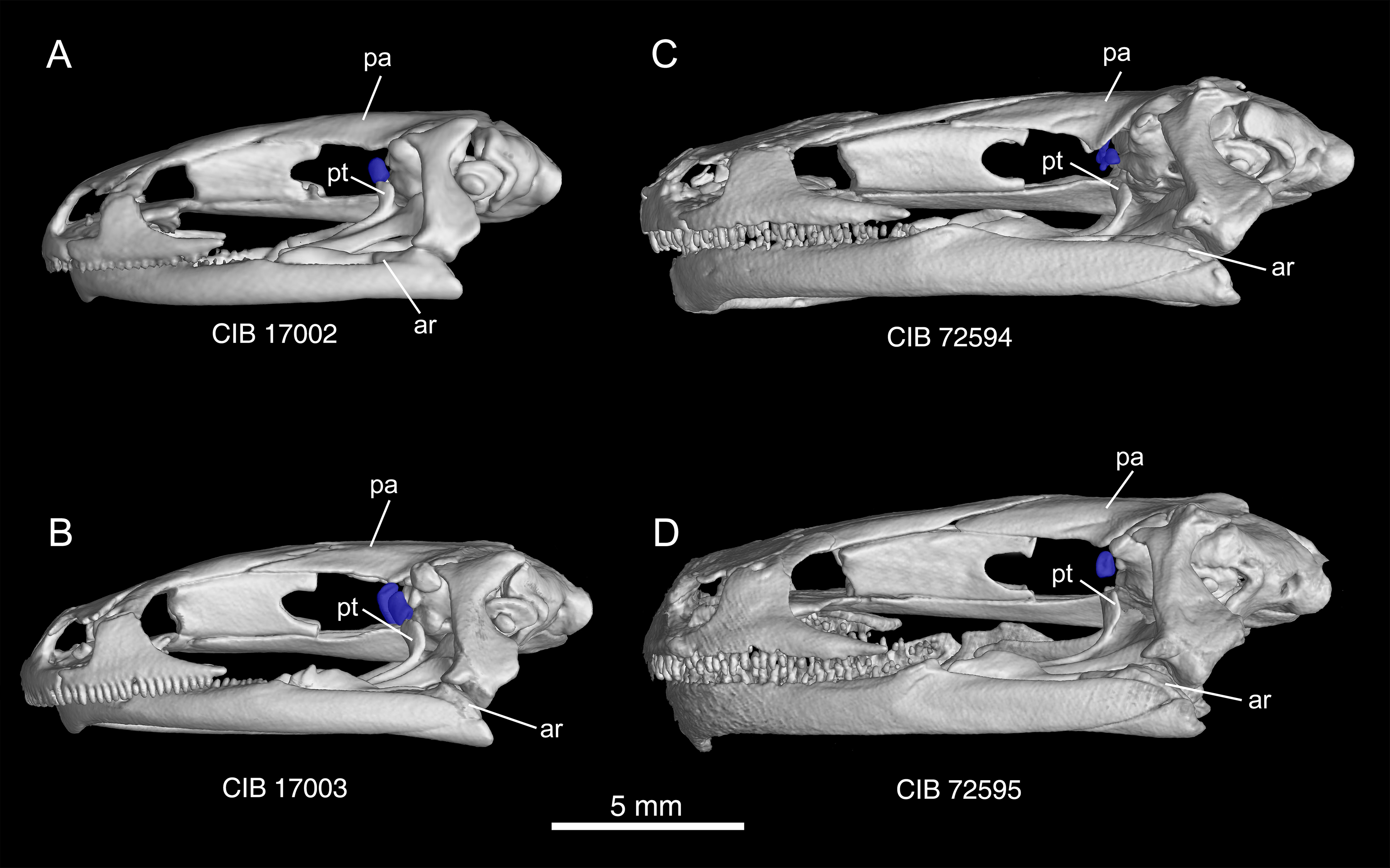

Supplement: S7 Fig — A, CIB 17002; B, CIB 17003; C, CIB 72594; D, CIB 72595. CIB 17002, and 17003 from Shenguozhuang, Yuexi County; CIB 72594, and 72595 from Tuowu, Mianning County. (TIF) [file pone.0211069.s007.tif]
