# Supplementary material for: Osteology of Batrachuperus yenyuanensis (Urodela, Hynobiidae), a high-altitude mountain stream salamander from western China
Source: PLoS One. 2019 Jan 25;14(1):e0211069. doi: 10.1371/journal.pone.0211069 (PMC6347256; doi:10.1371/journal.pone.0211069)
Supplement: S1 Table — (DOCX) [file pone.0211069.s008.docx]

S1 Table. Information on specimens used in this study

(All measurements in millimeters; elevation in meters)

| Catalogue Number | Total Length  (mm) | Snout-Pelvic  Length (mm) | Skull Length  (mm) | Skull Width  (mm) | Sex | Date | Juvenile/  Adult/  Subadult^*^ | Locality | Coordinates | | Elevation  (m) |
| --- | --- | --- | --- | --- | --- | --- | --- | --- | --- | --- | --- |
| CIB 14514 | 187.66 | 98.42 | 17.26 | 13.36 | ♀ | 1965 | Subadult | Maoniudui | N27°60′995′′ | E102°32′502′′ | 3650 |
| CIB 14548 | 157.18 | 80 | 15.50 | 12.27 | ♀ |  | Subadult |  |  |  |  |
| CIB 14550 | 183.58 | 91.84 | 13.05 | 10.11 | ♂ |  | Adult |  |  |  |  |
| CIB 16999 | 181.16 | 89.76 | 17.2 | 13.98 |  | 1965 | Subadult | Shenguozhuang | N28°32′002′′ | E102°45′659′′ | 2970 |
| CIB 17002 | 133.82 | 73.24 | 13.95 | 9.73 |  |  | Subadult |  |  |  |  |
| CIB 17003 | 144.64 | 76.96 | 14.85 | 11.21 | ♂ |  | Adult |  |  |  |  |
| CIB 17005 | 183 | 93 | 17.65 | 13.73 |  |  | Subadult |  |  |  |  |
| CIB 17302 | 163.35 | 76.99 | 16.05 | 12.58 |  | 1942 | Subadult | Shuangertang  (Bailinshan  in other references) | N27°20′319′′ | E101°32′253′′ | 2850 |
| CIB 17305 | 172.65 | 82.37 | 15.75 | 12.68 |  | 1942 | Subadult |  |  |  | 2850 |
| CIB17307 | 152.9 | 72.28 | 14.67 | 12.13 |  | 1942 | Subadult |  |  |  | 2850 |
| CIB 17308 | 201.09 | 90.31 | 17.60 | 13.00 |  | 1942 | Adult |  |  |  | 2850 |
| CIB 17309 | 160.05 | 77.37 | 15.15 | 11.40 |  | 1942 | Subadult |  |  |  | 2850 |
| CIB 17310 | 173.13 | 83.34 | 20.30 | 15.11 |  | 1942 | Adult |  |  |  | 2850 |
| CIB 17313 | 169.37 | 82.27 | 16.67 | 13.12 |  | 1942 | Subadult |  |  |  | 2850 |
| CIB 17314 | 158.68 + | 83.06 | 15.30 | 11.26 | ♀ | 1942 | Subadult |  |  |  | 2850 |
| CIB 2010072723 | 148.22 | 79.67 | 13.45 | 10.22 |  | 2010 | Subadult |  |  |  | 2850 |
| CIB 201707YY01 | 140.46 | 80.87 | 14.66 | 11.82 |  | 2017 | Subadult |  |  |  | 3200 |
| CIB 201707YY02 | 145.45 | 84.24 | 13.21 | 10.90 |  | 2017 | Subadult |  |  |  | 3200 |
| CIB 201707YY04 | 113.34 | 63.48 | 16.36 | 12.76 | ♂ | 2017 | Subadult |  |  |  | 3200 |
| CIB 201707YY08 | 169.48 | 88.72 | 21.32 | 16.58 | ♂ | 2017 | Adult |  |  |  | 3200 |
| CIB 201707YY09 | 154.78 | 84.32 | 19.22 | 15.88 | ♀ | 2017 | Subadult |  |  |  | 3200 |
| FMNH 49371 | 174.8 | 80.32 | 15.89 | 11.47 |  | 1942 | Subadult |  |  |  | 4025 |
| CIB 72592 | 174.96 | 91.47 | 16.89 | 13.52 |  | 1986 | Adult | Tuowu | N28°57′028′′ | E102°17′784′′ | 2500 |
| CIB 72593 | 170.13 | 86.2 | 15.33 | 12.57 |  |  | Adult |  |  |  |  |
| CIB 72594 | 195.83 | 89.34 | 18.86 | 14.37 |  |  | Adult |  |  |  |  |
| CIB 72595 | 197.28 | 91.58 | 18.52 | 13.55 |  |  | Adult |  |  |  |  |
| CIB 72596 | 181.08 | 85.23 | 16.55 | 12.38 |  |  | Adult |  |  |  |  |
| CIB 72597 | 196.83 | 92.31 | 19.12 | 13.60 |  |  | Adult |  |  |  |  |
| CIB 72598 | 174.59 | 89.53 | 16.80 | 13.40 |  |  | Adult |  |  |  |  |
| CIB 72599 | 231.39 | 111.81 | 19.4 | 16.98 |  |  | Adult |  |  |  |  |
| CIB 88795 | 142.38 | 75.42 | 13.46 | 10.72 |  | 1984 | Subadult | Heilongtan | N27°58′176′′ | E102°37′973′′ | 3650 |
| CIB 88799 | 110.52 | 58.96 | 12.55 | 10.06 |  |  | Subadult |  |  |  |  |
| CIB 88803 | 120 | 64.56 | 12.85 | 9.45 |  |  | Subadult |  |  |  |  |
| CIB 88815 | 90.8 | 48 | 11.05 | 8.57 |  |  | Juvenile |  |  |  |  |
| CIB 94627 | 132.1 | 75.96 | 14.26 | 11.61 |  |  | Juvenile | Xieka | N28°55′646′′ | E101°50′336′′ | 3116 |
| CIB 94631 | 155.48 | 82.72 | 17.05 | 14.21 |  | 2006 | Subadult |  |  |  |  |
| CIB 94632 | 153.6 | 78.42 | 15.6 | 12.41 |  |  | Subadult |  |  |  |  |

* Note: Adults are characterized by fully ossified articular and mesopodial elements; subadults refer to those specimens with an incomplete ossification of articular and mesopodial elements; juveniles are post-metamorphic individuals lacking any ossification of articular, without or limited ossification of the mesopodial elements.
