# Supplementary material for: Osteology of Batrachuperus yenyuanensis (Urodela, Hynobiidae), a high-altitude mountain stream salamander from western China
Source: PLoS One. 2019 Jan 25;14(1):e0211069. doi: 10.1371/journal.pone.0211069 (PMC6347256; doi:10.1371/journal.pone.0211069)
Supplement: S2 Table — (DOCX) [file pone.0211069.s009.docx]

S2 Table. Parameters of Micro-CT Scanning of specimens used in this study

| Catalogue Number | Current  (μA) | Voltage (kV) | Slices | Section # | Image Resolution  (pixels) | Voxel Size  (μm) | CT Scanner | Date of Scan |
| --- | --- | --- | --- | --- | --- | --- | --- | --- |
| CIB 14514 | 60 | 90 | 512/section | 7 | 512 | 36.9–90 | Quantum | 2017/11/04 |
| CIB 14548 |  |  |  | 6 |  | 33.3–90 |  | 2017/11/05 |
| CIB 14550 |  |  |  | 8 |  | 37.7–90 |  | 2017/11/04 |
| CIB 16999 | 60 | 90 | 512/section | 7 | 512 | 37.4–90 | Quantum | 2017/11/04 |
| CIB 17002 |  |  |  | 6 |  | 32.4–90 |  | 2017/11/05 |
| CIB 17003 |  |  |  | 6 |  | 33.5–90 |  | 2017/11/05 |
| CIB 17005 |  |  |  | 7 |  | 37.5–90 |  | 2017/11/04 |
| CIB 17302 | 88 | 90 | 512/section | 7 | 512 | 50 | Quantum | 2017/06/15 |
| CIB 17305 |  |  |  | 8 |  | 38.3–50 |  | 2017/06/15 |
| CIB17307 |  |  |  | 7 |  | 38.4–50 |  | 2017/06/16 |
| CIB 17308 | 88 | 90 | 512/section | 9 | 512 | 46.8–50 | Quantum | 2017/06/14 |
| CIB 17309 |  |  |  | 8 |  | 50 |  | 2017/06/15 |
| CIB 17310 |  |  |  | 8 |  | 41.6–50 |  | 2017/06/15 |
| CIB 17313 |  |  |  | 7 |  | 50–90 |  | 2017/06/13 |
| CIB 17314 | 88 | 90 | 512/section | 7 | 512 | 50 | Quantum | 2017/06/14 |
| CIB 2010072723 | 88 |  |  | 5 |  | 40.9–90 |  | 2017/03/31 |
| CIB 201707YY01 | 60 |  |  | 6 |  | 36.6–90 |  | 2017/07/21 |
| CIB 201707YY02 | 60 | 90 | 512/section | 6 | 512 | 38.3–90 | Quantum | 2017/07/21 |
| FMNH 49371 | 195 | 140 | 2000/section | 4 | 2000 | 33.336 | GE v/tome/x | 2016/10/12 |
| CIB 72592 | 88 | 90 | 512/section | 7 | 512 | 41.6–90 | Quantum | 2017/06/21 |
| CIB 72593 | 88 | 90 | 512/section | 7 | 512 | 41–90 | Quantum | 2017/06/22 |
| CIB 72594 | 88 | 90 | 512/section | 7 | 512 | 50–90 | Quantum | 2017/06/20 |
| CIB 72595 |  |  |  | 9 |  | 41.7–90 |  | 2017/06/21 |
| CIB 72596 |  |  |  | 7 |  | 40.4–90 |  | 2017/06/21 |
| CIB 72597 | 88 | 90 | 512/section | 7 | 512 | 40.8–90 | Quantum | 2017/06/21 |
| CIB 72598 |  |  |  | 7 |  | 40–90 |  | 2017/06/20 |
| CIB 72599 |  |  |  | 8 |  | 72–90 |  | 2017/06/20 |
| CIB 88795 |  |  |  | 5 |  | 31.8–90 |  | 2017/11/05 |
| CIB 88799 | 88 | 90 | 512/section | 5 | 512 | 27.7–90 | Quantum | 2017/11/05 |
| CIB 88803 |  |  |  | 5 |  | 28.6–90 |  | 2017/11/05 |
| CIB 88815 |  |  |  | 5 |  | 25–50 |  | 2017/11/05 |
| CIB 94627 | 88 | 90 | 512/section | 5 | 512 | 32.3–90 | Quantum | 2017/11/06 |
| CIB 94631 |  |  |  | 6 |  | 33–90 |  | 2017/11/05 |
| CIB 94632 |  |  |  | 6 |  | 34.2–90 |  | 2017/11/06 |
